# Thermal rectification in multilayer phase change material structures for energy storage applications

**DOI:** 10.1016/j.isci.2021.102843

**Published:** 2021-07-10

**Authors:** Timm Swoboda, Katja Klinar, Shahzaib Abbasi, Gerrit Brem, Andrej Kitanovski, Miguel Muñoz Rojo

**Affiliations:** 1 Department of Thermal and Fluid Engineering, University of Twente, Enschede, Overijssel 7500, The Netherlands; 2 Faculty of Mechanical Engineering, University of Ljubljana, Osrednjeslovenska, Askerceva 6, 1000, Ljubljana, Slovenia

**Keywords:** energy engineering, materials science, energy materials

## Abstract

Solid-state thermal control devices that present an asymmetric heat flow depending on thermal bias directionality, referred to as thermal diodes, have recently received increased attention for energy management. The use of materials that can change phase is a common approach to design thermal diodes, but typical sizes, moderate rectification ratios, and narrow thermal tunability limit their potential applications. In this work, we propose a multilayer thermal diode made of a combination of phase change and invariant materials. This device presents state-of-the-art thermal rectification ratios up to 136% for a temperature range between 300 K and 500 K. Importantly, this design allows to switch between distinct rectification states that can be modulated with temperature, achieving an additional degree of thermal control compared with single-rectification-state devices. We analyze the relevance of our thermal diodes for retaining heat more efficiently in thermal storage elements.

## Highlights

• Unique thermal diode design based on multilayer phase change material structures
• Thermal rectification ratios up to 136 % were observed
• The thermal rectification ratio can be modulated with temperature
• Thermal diodes can be integrated in energy storage elements for efficient heat retention

## Graphical abstract

**Figure:**
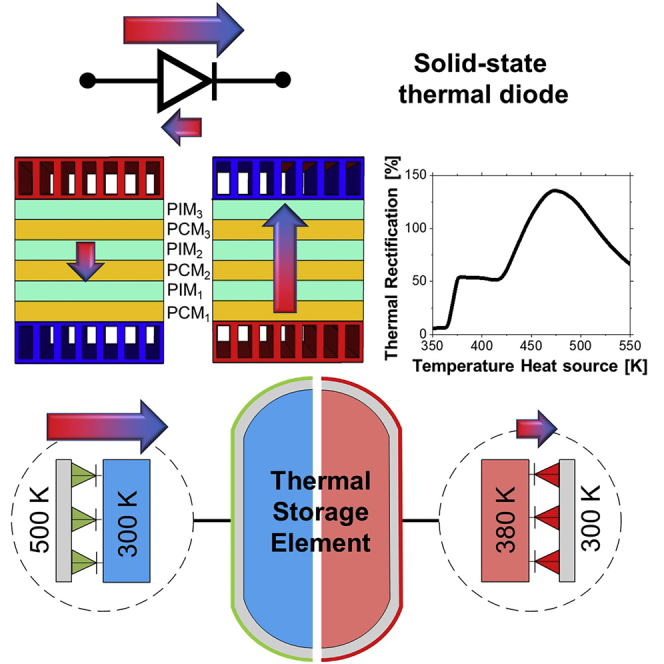

## Introduction

In recent years, solid-state thermal control devices (Swoboda et al., 2020) have received increased attention as a potential solution for improved energy management, storage, or conversion (Klinar et al., 2020b; Liu et al., 2019; Swoboda et al., 2020; Wong et al., 2021). Thermal diodes, regulators, switches, or transistors are capable of managing heat in a manner analogous to how electricity is controlled by their electrical counterparts (Wehmeyer et al., 2017). Among this toolkit of richer thermal control elements, thermal diodes have received special attention for their unique opportunities in solar thermal energy harvesting (Wehmeyer et al., 2017), caloric refrigeration (Klinar et al., 2020a; Wehmeyer et al., 2017), or in thermoelectric modules (Ghoshal and Guha, 2009; Wehmeyer et al., 2017). In thermal diodes, also referred to as thermal rectifiers, the heat flows preferably in one direction, resulting in an asymmetric transfer function (Li et al., 2004; Peyrard, 2006; Starr, 1936). The performance of a thermal diode is usually determined by the rectification ratio (*RR*),

(Equation 1) $$RR=\frac{\left|q_{fwd}\right|-\left|q_{rev}\right|}{\left|q_{rev}\right|}\cdot 100\%$$

where $q_{fwd}$ and $q_{rev}$ are heat fluxes in the forward (i.e., considered as the higher magnitude of heat flux) and reverse direction (i.e., lower magnitude of heat flux), respectively, when an equal temperature gradient along the device is considered in both directions.

Current approaches to develop solid-state devices with high thermal *RR*s are typically based on material engineering (Chang et al., 2006; Jiang et al., 2010; Li et al., 2012; Muñoz Rojo et al., 2019; Sawaki et al., 2011; Schmotz et al., 2011; Tian et al., 2012; Wang et al., 2017; Wu and Li, 2007; Yang et al., 2008, Yang et al., 2009; Zhu et al., 2014) or on a combination of materials with dissimilar properties. Among the materials proposed, phase change materials with a solid to solid phase transition (PCMs) are one of the most popular approaches for designing a thermal diode (Fiorino et al., 2018; Hamaoui et al., 2019; Ito et al., 2014; Kobayashi et al., 2012). Some PCMs present a phase transition at a critical temperature (*T*_*trans*_), which leads to a change in the thermal conductivity of the material. As an example, VO_2_ (Bohaichuk et al., 2019a; Bohaichuk et al., 2019b) is a well-known PCM, widely used for the development of smart windows (Manning et al., 2002) (Gao et al., 2012) and electronic devices (Bohaichuk et al., 2020; Zhou and Ramanathan, 2015), that has been studied as a thermal rectifier (Fiorino et al., 2018; Ito et al., 2014; Ordonez-Miranda et al. 2018). It typically presents a phase change transition at T∼340 K (Stefanovich et al. 2000) that results in a change of the lattice structure, from a monoclinic insulating to a tetragonal metallic phase (Eyert, 2002; Zhang et al., 2018). While dynamic changes in the structure of materials are very attractive to develop thermal diodes, the thermal conductivity variation in PCMs is typically moderate (Oh et al., 2010). Alternatively, PCMs such as VO_2_ can be combined with a phase invariant material (PIM), such as Si, to enhance their thermal rectification properties by inducing larger asymmetry in the heat flux across this structure (Fiorino et al., 2018; Ito et al., 2014; Kobayashi et al., 2012)

**Figure 1:**
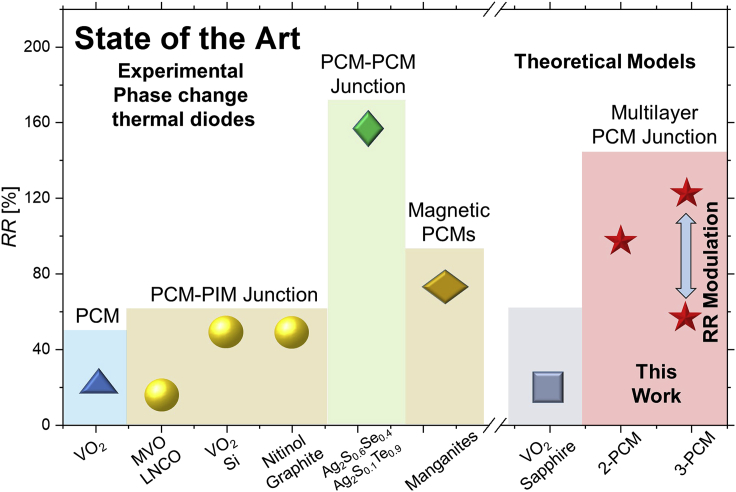
State-of-the-art State-of-the-art thermal rectification ratio of some of the most relevant thermal diodes - experimental and theoretical values - and the results of our proposed multilayer diodes (2-PCM diode based on PCM_1_: Ag_2_Te - PIM_*1*_: SiO_*2*_ - PCM_2_: Ag_*2*_S_*0.6*_Se_*0.4*_ - PIM_*2*_: Si; 3-PCM diode based on PCM_*1*_: Ag_*2*_S_*0.6*_Se_*0.4*_ - PIM_*1*_: Si PCM_*2*_: Ag_*2*_S_*0.8*_Se_*0.2*_ - PIM_*2*_: SiO_*2*_ - PCM_*3*_: Ag_*2*_Te - PIM_*3*_: Si) (Fiorino et al., 2018; Garcia-Garcia and Alvarez-Quintana, 2014; Hirata et al., 2020; Kobayashi et al., 2012; Martinez-Flores et al., 2018; Ordonez-Miranda et al., 2018; Zhu et al., 2014). For more information about other theoretical and experimental types of thermal diodes based on different operating principles, we would like to refer to the review article (Swoboda et al., 2020).

Figure 1 shows an overview of the rectification values obtained in the field of PCM thermal rectification and the obtained *RR* values of the diode design proposed in this work. On the one hand, a *RR* of up to *RR =* 28% has been observed in a pure PCM structure made of a VO_2_ beam with an asymmetric shape (Zhu et al., 2014). On the other hand, higher *RR*s of up to *RR* = 50% have been observed by combining PCMs such as VO_2_ (Fiorino et al., 2018; Hamaoui et al., 2019) or Nitinol(Garcia-Garcia and Alvarez-Quintana, 2014) with a PIM. Other examples include MVO (MnV_2_O_4_, PCM) and LNCO (La_1.98_Nd_0.02_CuO_4_, PIM) structures that present a thermal *RR* of 14% at low temperatures (∼55 K) (Kobayashi et al., 2012). Additionally, the combination of two different PCMs is promising for potentially higher thermal rectification values (Hirata et al., 2020; Kang and Yang, 2018; Leon-Gil et al., 2019). More specifically, a thermal diode with *RR* = 170% was found by combining two PCMs with an increase (Ag_2_S_0.6_Se_0.4_) and a decrease (Ag_2_S_0.1_Te_0.9_) in thermal conductivity with temperature (Hirata et al., 2020). All these efforts aim to the development of high-*RR* thermal diodes that bring promising opportunities for thermal management in electronics (Alto University, 2012; Cheh and Zhao, 2012; Roberts and Walker, 2011; Zhang and Zhang, 2011), caloric refrigeration (Klinar et al., 2020a; Wehmeyer et al., 2017), or new thermal technology, like heat logic (Sklan, 2015).

In this work, we report a distinct approach toward the design of realistic and experimentally achievable thermal diodes using multilayer structures based on PCMs and PIMs. This approach combines the strategies used in PCM-PIM and PCM-PCM devices to develop a novel thermal diode design with high performance and distinct rectification states for advanced thermal control. First, a finite element model (FEM) is used to evaluate the optimum design configuration that maximizes the thermal rectification in these structures. Using this tool, we evaluate different geometries (2-PCM/PIM vs 3-PCM/PIM diodes), temperature ranges, and material configurations. Then, we calculate the thermal *RR* to be 106% and 136% for the 2-PCM/PIM and 3-PCM/PIM structures for Δ*T* = 95 K and Δ*T* = 172 K, respectively (Δ*T* = *T*_*source*_ – *T*_*sink*_). Our approach shows a functional, adjustable thermal diode design, which can be applied for various material configurations. Additionally, we observe that the rectification properties of our 3-PCM/PIM can be modulated between an intermediate *RR* to a high *RR* state depending on the thermal bias Δ*T*. This feature shows a clear difference from previously reported single-rectification-state thermal diodes and enables another degree of freedom for thermal management applications. Finally, we determine the ability of this diode to retain energy when it is integrated in thermal storage elements.

### Modeling approach

**Figure 2:**
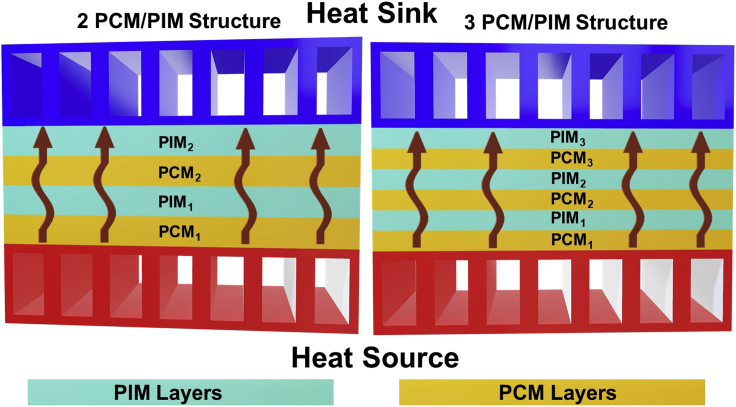
Schematic drawing of the two-diode configurations (left 2-PCM/PIM diode, right 3-PCM/PIM diode) proposed in this work. The arrows indicate the heat flux direction from the heat source to the heat sink.

The proposed design of the thermal diode consists of a stack of multiple PCM and PIM layers in an alternating configuration (see Figure S1). This design expands current configurations of PCM and PIM diode structures, which are mentioned in the introduction (Fiorino et al., 2018); Garcia-Garcia and Alvarez-Quintana, 2014; Ito et al., 2014; Kobayashi et al., 2012; Ordonez-Miranda et al., 2018). First, we analyze the *RR* with two PCM and PIM layers (2-PCM/PIM diode) and then we extend it to three PCM and PIM layers (3-PCM/PIM diode). Figure 2 illustrates the two designs. These diodes specially aim to not only enhance the rectification ratio by using distinct multilayer structures but also develop a device that is experimentally achievable to warrant its use for energy management applications.

The performance of these thermal diodes is analyzed through FEM, using COMSOL Multiphysics. A two-dimensional thermal transport model analyzes the conductive heat flux across the structures depending on directionality (see Section S3). Before carrying out simulations in our proposed diodes, we first validate our model on base of a PCM/PIM thermal diode design proposed in a prior study in literature (Ordonez-Miranda et al., 2018) (see Section S7). In their study, the thermal *RR* of a VO_2_ (PCM) and sapphire (PIM) thermal diode is calculated theoretically. We use our COMSOL simulation to calculate the *RR* of this diode using their exact same geometry, material configuration, and thermal bias (see Figure S7). We obtain a maximum *RR* = 20.1%, which match well with their results (*RR* = 19.7%).

After validation, we use this COMSOL model to analyze the thermal transport in our 2-PCM/PIM and 3-PCM/PIM diodes. First, we fix the temperature of the heat sink (cold side), *T*_*sink*_, at 300 K while varying the temperature of the heat source (hot side), *T*_*source*_, between 300 K and 550 K (see Figure S3). The position of the heat sink and source is exchanged to investigate the thermal rectification effect depending on the temperature gradient directionality, i.e., forward vs reverse heat flow. The structures are assumed to be thermally insulated at the edges; thus, potential heat losses by convection or radiation with the surrounding environment are not included (see Figure S3). For the simulations, a predefined mesh is used (see Figure S4). Then, the heat flux, *q,* across the structure is calculated in steady state for both cases using Fourier 's law,

(Equation 2) q=−k⋅∇T

where *k* is the thermal conductivity of the material and ∇*T* the temperature gradient across the material.

For the thermal conductivity of the different PCM and PIM layers used in our models, we interpolate experimental values from literature (Chen et al., 2018; Hirata et al., 2020; Oh et al., 2010) (see Table S1 and Figure S2). PCMs can be classified according to their type of change in their thermal conductivities over their respective transition temperatures. We define type A and type B PCMs as those that exhibit increased and decreased thermal conductivity above the transition temperature, respectively (see Table S1). In both configurations, type A and type B PCMs are combined in an order such that the PCMs are exclusively in their high conductive state in the forward direction while being in the low conductive state in the reverse direction. For the PIM layers, we consider a higher conductive material (silicon) and a lower conductive material (SiO_2_) which are predefined in the COMSOL database (see Section S2). The temperature drop in the PIM layers enables a better combination of PCMs with different phase transition temperatures. As a result of that, we make sure that each PCM possesses the ideal transition temperature in the applied temperature range. Each possible material combination under the predefined configuration is evaluated (see Figure S2). The total length, *l*, and width, *w*, of the structure is set to 1.2 μm (see Figure S1). The structures are modeled using individual rectangular-shaped polygons, in which the length of the individual layers is distributed equally at a constant width (see Section S1).

Additionally, in this model, we also account for thermal interface resistances (see Figure S3). According to Lyeo and Cahill, 2006, the thermal conductance at the border of two dissimilar materials, i.e. the thermal interface conductance *G*_*interf*_ falls within a narrow range of values. Based on their results (Lyeo and Cahill, 2006), we approximate the thermal interfacial conductance between layers to be ∼100 MW/(m^2^·K), which is equal to a thermal interface resistance *R*_*interf*_ of ∼10^−8^ (m^2^·K)/W. The number of interfaces between blocks, *n*_*interf*_*,* is 3 in the case of the 2-PCM/PIM diodes and 5 in the case of the 3-PCM/PIM diode structure. Later in this article, we discuss how changes in the thermal interface resistances possibly affect the result of the thermal rectification.

### Results of the PCM/PIM thermal diode

In this section, the thermal *RR*s for the thermal diodes illustrated previously under stationary conditions are presented. We show first the results of the 2-PCM/PIM multilayer structure followed by those obtained for 3-PCM/PIM. In each case, we determine the conductive heat flux and temperature profile of the structure (See Section S5). As we treat the diode configuration as a series of resistances, we expect to observe a constant heat flux along the structure (see Figure S5). We calculate the *RR* by means of Equation 1. For each diode design, we analyze the impact of the different parameters, such as thermal bias and the material configuration, on the thermal rectification properties.

#### RR of 2-PCM/PIM & 3-PCM/PIM multilayers for a thermal bias of ΔT = 200K

**Figure 3:**
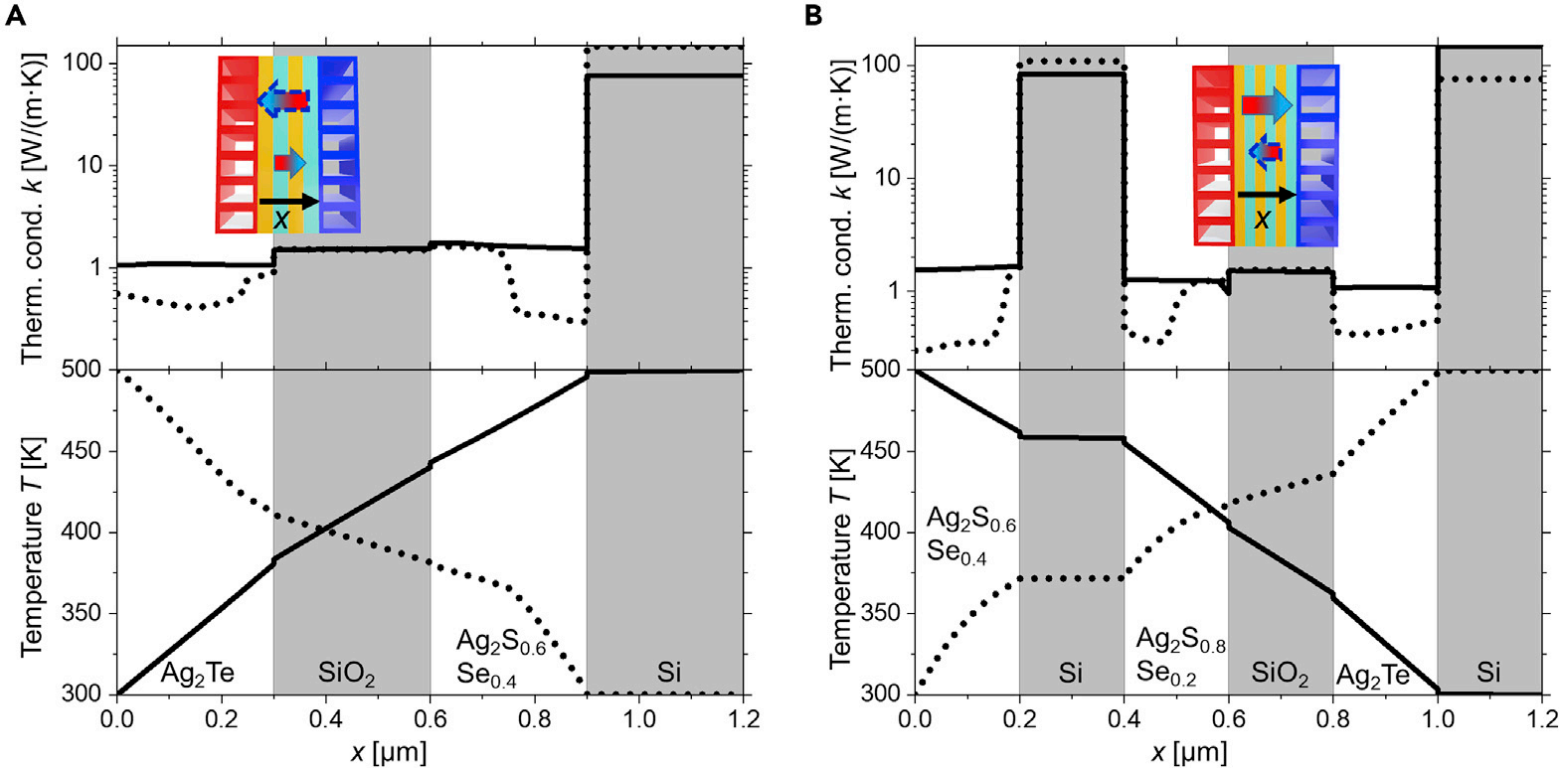
Thermal diode Thermal conductivity and temperature profile of the (A) 2-PCM/PIM structure as a function of *x,* as indicated in the inset drawing and; (B) 3-PCM/PIM structure as a function of *x* as indicated in the inset drawing. The solid and dotted lines correspond to the forward and reverse direction, respectively. The dotted arrows in the inset drawings present the heat flux magnitudes when the temperature reservoirs are exchanged. The directions with preferred heat transfer are indicated by larger gradient arrows in the inset drawings. The inset drawings show that the arrows are reversed for the 2-PCM/PIM vs 3-PCM/PIM designs, which is due to the new material layers and their differences in phase change transition temperatures.

For the 2-PCM/PIM multilayer structure, we use the material reported data in Section S2 to determine the ideal material combinations that maximize the rectification performance of this diode. For a thermal bias of Δ*T* = 200 K (heat source and sink at 500 K and 300 K, respectively), the highest *RR* ≈ 96% is obtained for the following 4-layer material configuration: PCM_1_: Ag_2_Te - PIM_*1*_: SiO_*2*_ - PCM_2_: Ag_*2*_S_*0.6*_Se_*0.4*_ - PIM_*2*_: Si. The effective thermal conductivity in the reverse and forward direction is *k*_*eff,rev*_ ≈ 0.89 W/(m·K) and *k*_*eff,fwd*_ ≈ 1.74 W/(m·K), respectively (see Section S6). Figure 3A) presents the temperature profiles across the structure and thermal conductivities of each layer in the forward and reverse directions for the 2-PCM/PIM diode.

In Figure 3A), when the heat flows from right to left in the inset figure, the temperature gradient across the two PCMs is above their phase transition temperature, leading to low thermal resistance (PCMs with high thermal conductivity – forward direction). When the heat flow is reversed (left to right), the temperature gradient across the PCM is mainly below the phase transition temperature, resulting in higher thermal resistance phases (PCMs with lower thermal conductivity – reverse direction). Apart from that, the PIMs, Si or SiO_2_, present almost negligible variation in its thermal conductivity. In Figure 3A, the temperature drop across the PCM and PIM materials is plotted. In the forward direction (PCM_1_:Ag_2_Te at low temperatures), the temperature drop in SiO_*2*_ warrants that the temperature in PCM_*1*_ Ag_*2*_Te is below its transition temperature across the whole layer. In the reverse direction, the temperature drop in the SiO_*2*_ layer enables the temperature in PCM_*2*_ Ag_*2*_S_*0.6*_S_*0.4*_ to fall below the phase transition temperature. Therefore, the PIM layers allow a better control of the transition temperatures for the PCM layers involved.

Similarly, as for the 2-PCM/PIM diode, we use reported data to determine the thermal *RR* in a 3-PCM/PIM multilayer diode configuration (see Section S2). The highest *RR* for a thermal bias of Δ*T* = 200 K (heat source and sink at 500 K and 300 K, respectively) is found to be *RR* ≈ 119%, corresponding to the following 6-layer configuration: PCM_*1*_: Ag_*2*_S_*0.6*_Se_*0.4*_ - PIM_*1*_: Si PCM_*2*_: Ag_*2*_S_*0.8*_Se_*0.2*_ - PIM_*2*_: SiO_*2*_ - PCM_*3*_: Ag_*2*_Te - PIM_*3*_: Si. The effective thermal conductivity in the reverse and forward directions is *k*_*eff,rev*_ ≈ 0.83 W/(m·K) and *k*_*eff,fwd*_ ≈ 1.82 W/(m·K), respectively (see Section S6). Figure 3B presents the temperature gradient across the diode and the thermal conductivity values of each layer. On the one hand, we can see that in the forward direction, when the heat flows from left to right in the inset figure, all the PCM materials are mostly in their high thermally conductive phases. On the other hand, all PCMs are in their low thermally conductive phases when the heat flow is reversed, except small segments in PCM_*1*_ and PCM_*2*_ that are in their high conductive states. If we compare these results with those presented for 2-PCM/PIM structure in Figure 3A, we observe that the number of PCM layers being in their low conductive phase in reverse direction for this temperature gradient is higher. Therefore, a 3-PCM/PIM multilayer structure provides a greater degree of thermal control than a 2-PCM/PIM structure.

#### RR of 2- & 3-PCM/PIM multilayers vs heat source temperature

**Figure 4:**
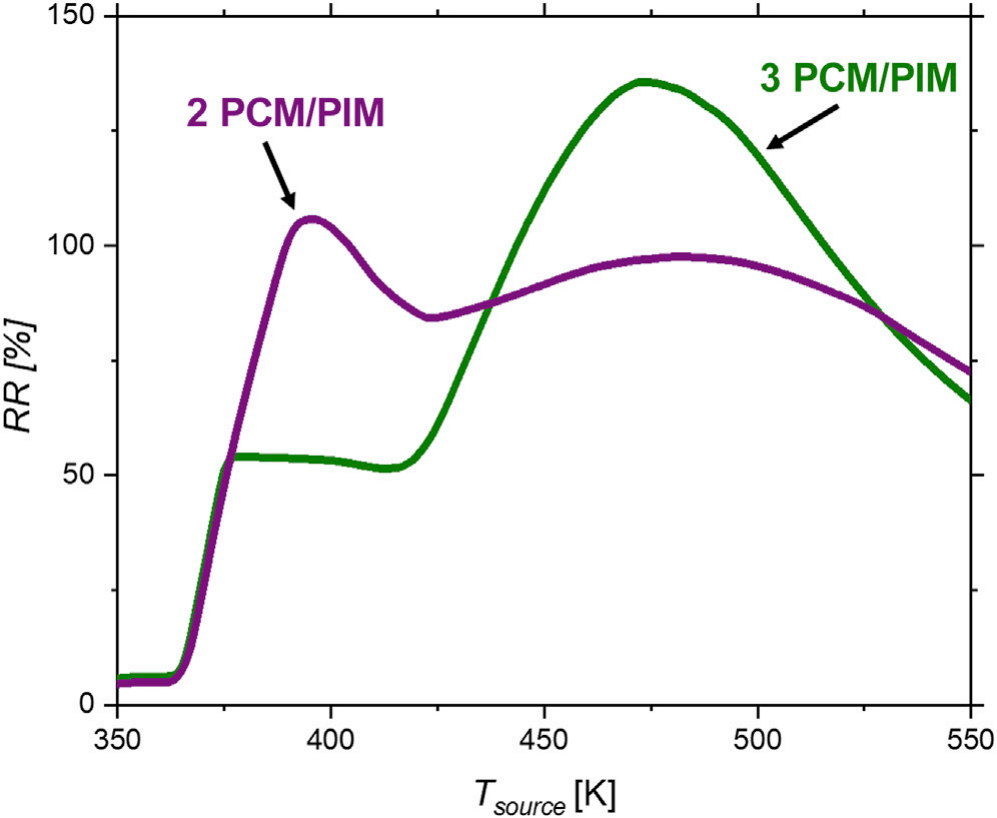
Thermal rectification ratio Thermal rectification ratio of 2-PCM/PIM (PCM_1_: Ag_2_Te - PIM_1_: SiO_2_ - PCM_2_: Ag_2_S_0.6_Se_0.4_ - PIM_2_: Si) and 3-PCM/PIM (PCM_1_: Ag_2_S_0.6_Se_0.4_ - PIM_1_: Si PCM_2_: Ag_2_S_0.8_Se_0.2_ - PIM_2_: SiO_2_ - PCM_3_: Ag_2_Te - PIM_3_: Si) multilayer structures as a function of the temperature of the heat source.

The *RR* of 2- and 3-PCM/PIM multilayer structures is also studied for different values of *T*_*source*_. For that purpose, we fix *T*_*sink*_ at 300 K and vary *T*_*source*_ (see Section S5). Figure 4 displays the results of the *RR* of both structures as a function of *T*_*source*_. In the 3-PCM/PIM multilayer structure, the highest *RR* ≈ 136% is found at *T*_*source*_ = 472 K, while for the 2-PCM/PIM structure, a maximum *RR* ≈ 106% is achieved at *T*_*source*_ = 395 K.

As can be seen in Figure 4, nearly no rectification can be observed around 350 K as the temperature of the structure is below the transition temperatures of the PCMs. The small values observed are due to temperature-dependent materials' thermal properties. In both structures, the *RR* rises for temperatures higher than *T*_*trans*_ of the common PCM block made of Ag_*2*_S_*0.6*_Se_*0.4*_ (*T*_*trans*_∼360 K). A higher *RR* is achieved for the 2-PCM/PIM multilayer structures for intermediate temperature ranges (375 K < T < 425 K). Differently, for temperatures between 425 K and 525 K, the 3-PCM/PIM multilayer structure leads to higher *RR*s. Finally, when the temperature becomes above ∼470 K, the *RR* drops as the temperature across PCMs remains above *T*_*trans*_ for both forward and reverse directions. Especially interesting is that the *RR* vs *T* dependency of the 3-PCM diode structure increases stepwise from an intermediate value for temperatures at around ∼400 K to higher values at higher temperature, reaching the peak at ∼470 K. This is related to a stepwise increase of the thermal conductivity in the forward direction (see Figure S6). For intermediate temperature, only PCM_1_ is in its high conductive state in the forward direction. When *T*_*source*_ is going above 425 K, PCM_2_ and PCM_3_ start to transition to its high conductive state up to the ideal configuration at around 470 K. As a result, this thermal diode shows promising properties as a multistate thermal regulator. A temperature-gated sequential thermal rectification effect has been previously reported by Martinez-Flores et al. (Martinez-Flores et al., 2018) in a manganite-based material system. However, our results distinguish from them as well as other ones on the fact that we use different materials, multiple segments, and two distinct rectification states that are stable in a certain temperature range. In the 2-PCM/PIM diode graph, the maximum rectification is reached at *T*_*source*_ = 395 K. At this point, PCM_2_ is completely in its low conductive state in the reverse direction. When increasing *T*_*source*_, PCM_2_ partially transitions to its high conductive state in the reverse direction and thus *RR* decreases until it reaches a minimum at 423 K. Above this temperature, PCM_1_ starts to transition to its low conductive state in the reverse direction and as a result *RR* rises again. PCM_1_ and PCM_2_ remain in their high conductive state in the forward direction for *T*_*source*_ > 380 K, so the changes in the *RR* are created owing to changes in the reverse configuration (see Figure S6A). We choose this temperature range because the used experimental data of the PCM materials do not present values above 550 K. However, we expect that *RR* will fall down to zero when further increasing *T*_*source*,_ as in these temperatures all PCMs will remain above *T*_*trans*_ in both directions.

#### Influence of geometry, interfaces, and the number of layers in thermal rectification of PCM/PIM diodes

The model developed in this work can also offer insights into the different design aspects of a PCM/PIM thermal diode. In this section, we discuss how the length, thermal interface, and the number of layers affect the thermal *RR* in this type of thermal diodes (see Section S8 Table S2). Additionally, we comment on feasibility related with the experimental fabrication of this diode and key parameters to take into account during this process.

As the length of the structure increases, in the presence of a fixed number of layers, the *RR* becomes larger because the influence of thermal interface resistance becomes smaller. If the thermal interface resistance becomes larger, the thermal rectification becomes smaller. As an example, if the thermal interface resistance is an order of magnitude higher (*R*_*interf*_ of ∼10^−7^ (m^2^·K)/W), the *RR* in the 3-PCM/PIM configuration becomes 83% instead of 119% when *T*_*source*_ = 500 K (see Table S2). The exact values of thermal interfacial resistance can be determined by applying the diffusive mismatch model (Swartz and Pohl, 1989). However, this requires precise information of the phonon dispersion relationship of the materials (Reddy et al., 2005). Nevertheless, an *R*_*interf*_ of ∼10^−7^ (m^2^·K)/W represents the upper limit of the interfacial resistance values (Lyeo and Cahill, 2006). On the one hand, we expect that the experimental value will be within these values. On the other hand, a lower interfacial resistance *R*_*interf*_ of ∼10^−9^ (m^2^·K)/W would lead to a slight increase of the *RR* of the aforementioned configuration to 126%.

When determining the *RR* of PCM/PIM multilayers, it is also important to consider the total number of PCM and PIM layers (see Table S2). As can be seen from the results presented in this study, the prediction of *RR* is dependent on the choice of materials as well as the total number of PCM/PIM layers. For the same diode size, the 3-PCM/PIM multilayer structure enables a higher degree of control of the thermal conduction phases in PCMs at specific temperature ranges than the 2-PCM/PIM structure. Therefore, one could argue that a 4-PCM/PIM multilayer structure could allow an even higher thermal control at higher temperatures. However, it is not necessarily the case because the PCM selection is limited to their *T*_*trans*_ values. The reason is that to optimize the diode performance, the PCM transition temperatures need to match with the temperature gradient across the segments added. Since the transition temperatures of the selected materials (Section S2 – see Table S1) are partly close to each other, having a combination of 4 PCMs is not bound to add much benefits compared with the 3-PCM/PIM structure under the same diode configuration. In this work, we use realistic values, reported in literature, for our PCMs. However, future material engineering approaches could lead to materials with tuned *T*_*trans*_ that enable optimum PCM/PIM multilayer thermal diodes with higher *RR*.

From the point of view of fabrication and measurements of the *RR* of this thermal diode, one should account for potential sources of discrepancy with this model. For example, the impact of heat losses due to radiation and convection under different working environments could lead to differences between the *RR* results observed in the model *vs* those measured. However, it has been reported that the thermal radiation energy density rather decreases at lower sizes (Yu et al., 2010). Moreover, the thermal properties at phase transition might vary depending on the quality of the PCM layers, the interface thermal resistance, as well as whether the PCM blocks transition phase completely or partially under certain temperature gradients across them. Finally, the thermal properties of the individual layers are also size dependent. Size confinement effects can lead to a reduction of the thermal conductivity values and the phase transition temperature and hysteresis (see Table S2) (Oh et al., 2010; Li et al., 2003).

### Discussion on thermal diode applications

In this section, we analyze the relevance of our thermal diodes based on our optimized PCM/PIM multilayer structures for energy storage applications. Owing to the desire to transition to an increasingly renewable energy infrastructure, energy storage is quickly becoming a key factor in designing and developing energy grids that are capable of withstanding the intermittent nature of most renewable energy sources. In terms of reducing carbon emissions and increasing the share of renewable energy sources on a larger scale, energy (or thermal) storage plays an important role (Henry et al., 2020). The intermittent and constantly changing power output of solar and wind power plants, which do not match the current energy demand, require energy storage. Nowadays, this is mostly done chemically with batteries, which is expensive and cannot guarantee long-term storage. With the development of more efficient power-to-x-to-power conversion, thermal storage has advantages in terms of lower cost, higher gravimetric and volumetric energy density, and being environmentally friendly.

**Figure 5:**
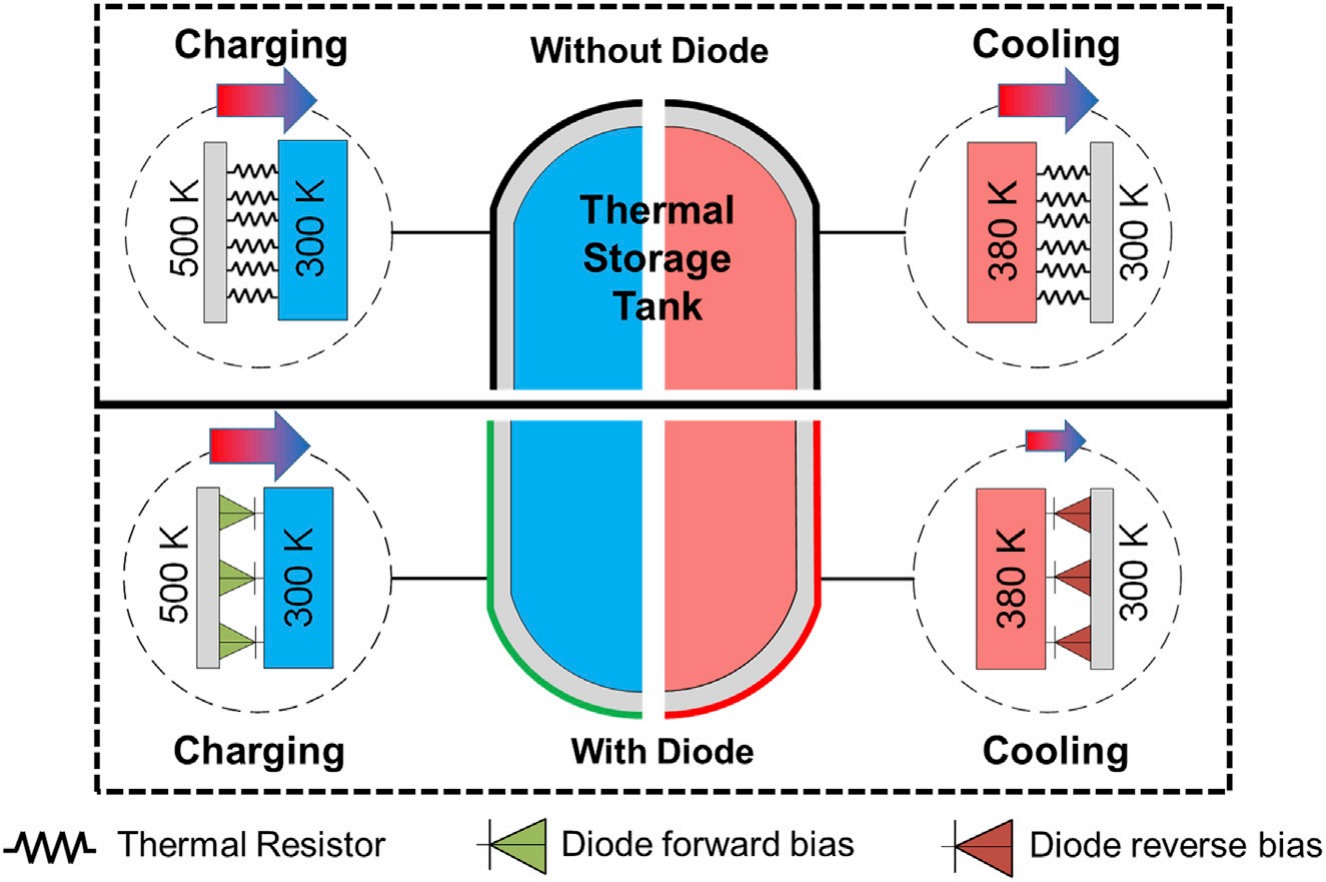
Schematic drawing of the heat fluxes from the energy storage element across the wall with and without the presence of PCM/PIM multilayer thermal diodes The area above the solid black line represents the situation without thermal diodes, while the area below shows the situation where the thermal diodes are integrated at the surface of the storage tank. In the left area (blue area in the thermal storage tank), the heat flows in during charging. In the right area (red area in the thermal storage tank), the heat flows out during cooling. The gradient arrows represent the magnitude of heat flux in both cases.

Within this context, we analyze thermal storage elements that represent intermediate ways to store surplus heat from fluctuating energy sources (e.g., from solar heat, industrial waste heat, conversion of surplus electricity) and utilize or convert it back when required. In this scenario, we consider an external liquid heat reservoir as an energy storage element that is contained in a shell onto which a thermal diode based on a PCM/PIM multilayer structure is attached. We analyze the processes of charging and cooling across the shell of the energy storage element with and without the presence of thermal diodes at the wall surface. During charging, the diodes are considered to operate in the forward direction and have lower thermal resistance, facilitating heat to be transported toward the storage element. When the heat source is not applied, the outer wall temperature of the PCM/PIM multilayer structure cools down, reversing the temperature gradient across this element. In the later scenario, the thermal diodes begin to transition to a higher thermal resistance, which allows better retention of heat inside the storage element. Figure 5 illustrates these two scenarios.

The thermal diodes that are on the wall of the energy storage element correspond to the 3-PCM/PIM multilayer structure presented in Figure 3B. In the analytical model, we consider a number of diodes, *n*_*diode*_*,* in parallel with an area, *A*_*diode*_*,* and thickness, *d*_*diode*_, matching the dimensions of the energy storage element. The thermal conductivities of the diode in the forward and reverse directions are calculated for all temperature gradients using our COMSOL model (see Figure S8). The temperature of the outer wall is assumed to be *T*_*source*_ = 500 K during the charging process and *T*_*sink*_ = 300 K in the cooling hours. We consider Paratherm NF as the heat storage medium with the reported mass *ρ*_*liquid*_ and the specific heat *c*_*liquid*_ (see Figure S8) (Paratherm, 2020). We separate the charging and cooling process as they are not applied simultaneously. Therefore, *t*_*ch*_ (0–6 hr) and *t*_*co*_ (0–49 hr) correspond to the time in the charging and cooling processes, respectively. The total run time *t* is then expressed as the sum of *t*_*ch*_ and *t*_*co*_. In the charging process, the initial temperature of the liquid is at room temperature *T*_*liquid*_ (*t*_*ch*_ = 0) = 300 K.

In our analytical model, we assume that heat transfer occurs only between the outer wall and the heat reservoir. Hence, the heat conduction is equal to the energy difference in the heated or cooled liquid. The behavior of the charging and cooling processes can be described based on previously mentioned parameters as follows:

(Equation 3) $$\text{Charging: }n_{diode}\cdot k_{fwd}\cdot A_{diode}\cdot \frac{T_{source}-T_{liquid}}{d_{diode}}=m_{liquid}\cdot c_{liquid}\cdot \frac{\delta \Delta T_{charge}}{\delta t_{ch}}$$

(Equation 4) $$\text{Cooling: }n_{diode}\cdot k_{rev}\cdot A_{diode}\cdot \frac{T_{liquid}-T_{sink}}{d_{diode}}=m_{liquid}\cdot c_{liquid}\cdot \frac{\delta \Delta T_{cooling}}{\delta t_{co}}$$

where$\;\Delta T_{charge}\left(t_{ch}\right)=T_{liquid}\left(t_{ch}\right)-\;T_{liquid}\left(t_{ch}=0\right)$ and $\Delta T_{cooling}\left(t_{co}\right)=\Delta T_{charge}\left(t_{1}\right)+T_{liquid}\left(t_{ch}=0\right)-T_{liquid}\left(t_{co}\right)$. The liquid temperature of the energy storage element is calculated with and without the presence of the diode. When no diode is present, we consider a material without rectification properties that presents the same thermal conductivity in the cooling mode as the one observed by the diode during the charging of the storage element (*k*_*rev*_ = *k*_*fwd*_). This results in no differences in the temperature for the cases with and without diode during the charging process.

First, we calculate the charging and cooling process for a situation in which the thermal storage element has thermal resistors on the shell wall (material with no thermal rectification). Second, we repeat the same process but replacing the thermal resistors with our thermal diodes. On the one hand, we calculate the temperature rise of the liquid, $\Delta T_{charge}$, during the charging process after a certain period of time (*t*_*1*_ *=* 6 hr; Δ*T*_*charge*_ [*t*_*1*_] = 79.7 K). On the other hand, during the cooling process, we calculate the temperature drop of the liquid, $\Delta T_{cooling}$, in the energy storage liquid. This is analyzed until we reach the initial temperature of the liquid using the non-diode configuration ($\Delta T_{cooling}\left(t_{2}\right)=\Delta T_{charge}\left(t_{1}\right)$); *t*_2_ ≈ 49 hr). Afterward, we calculate the temperature decrease for the same time span with the diode configuration. To simplify the denotation of the time- and temperature-independent parameters in our calculation, we introduce the following temperature-independent geometrical factor (*D*):

(Equation 5) $$D=\frac{n_{diode}A_{diode}}{d_{diode}\cdot V_{liquid}}$$

**Figure 6:**
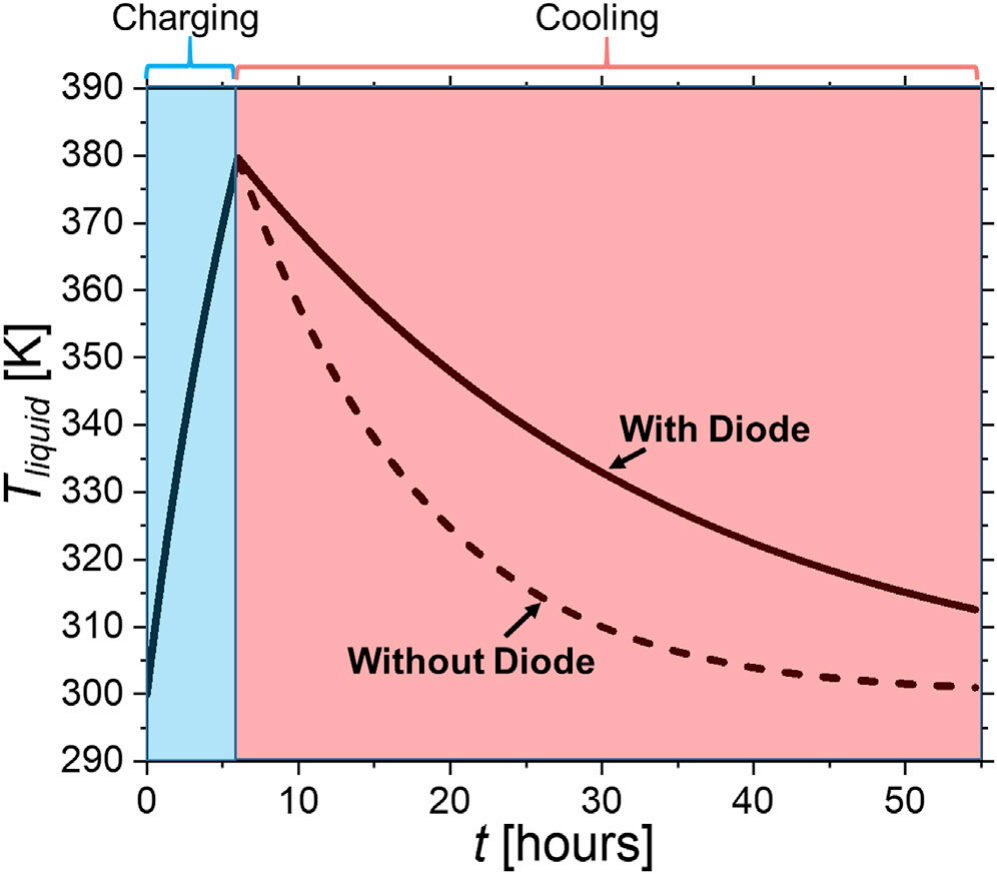
Temperature of the heat storage element, *T*_*liquid*_, as a function of time, *t,* during charging and cooling processes with (solid line) and without (dashed line) thermal diodes

The temperatures of the heat storage element during the charging and cooling processes are calculated for $D=23.53\;\frac{1}{m^{2}}$. For the geometrical parameters, we hypothesize the volume of the liquid to be *V*_*liquid*_ = 1.02 · 10^−6^ m^3^. Therefore, we assume a cubic liquid reservoir with a base length and width of 8 mm and a height of 16 mm. We choose *n*_*diode*_ = 20 that distribute over the flat surface of the tank wall, where each diode dimension is set to 1.2 μm for the width, length, and thickness. The mass of the system is calculated by multiplying the volume of the liquid by the density of the liquid ($m_{liquid}=\rho_{liquid}\cdot V_{liquid}=0.9\;g)$ (Paratherm, 2020). Figure 6 shows the calculated *T*_*liquid*_ based on the aforementioned approach and on the known material and diode properties. For the analytical model, we use polynomial fits for the diode and liquid properties (see Section S9 Table S3). In the non-diode configuration, we reach the original storage liquid temperature of ∼300 K after approximately 55 hr. For the same time span, the liquid temperature remains at ∼312.5 K for the diode configuration.

Finally, we calculate the magnitude of the total heat losses Δ*Q* during the cooling process with and without the diode. In the non-diode configuration, the heat liquid element heats up to 379.7 K. In the cooling process, the stored energy is transferred to the surroundings and the liquid cools down to its initial temperature of 300 K. The heat loss is calculated as the difference in the specific enthalpy states *h*_*1*_ at 300 K and *h*_*2*_ at 379.7 K (*Δh*) of the liquid, which we retrieve from literature (see Table S4) (Estimating Database – EES Data, n.d.). Thus, we can calculate the heat loss by using

(Equation 6) $$\Delta Q=\Delta h\cdot m_{liquid}$$

The charging process in the diode configuration is equal to the charging process in the non-diode configuration. In the cooling process, the liquid cools from 379.7 K to 312.5 K. Similar to the approach in the non-diode configuration, we determine the heat losses by calculating the differences in the enthalpy states *h*_*3*_ at 312.5 K and *h*_*2*_ at 379.7 K (Estimating Database – EES Data, n.d.). By using Equation 6, we obtain a heat loss of *ΔQ*_*no diode*_ = 148.5 J for the configuration without diode and *ΔQ*_*diode*_ = 127.3 J with diode. When we compare these results, we observe that the diode configuration is capable of retaining 17% more heat than in the case without diode. Moreover, we observe that the diode configuration enables an even better heat retention when its operation is considered for multiple day cycles (see Section S10). It is also important to mention that the experimental heat retention performance is further depending on the thermal contact between the hot/cold reservoirs and the diode (see Section S11). Fenech and Rohsenow, (1963). In this context, we observe that a low thermal contact resistance is required to maximize the heat retention to the prior mentioned value (see Section S11). We observe that a thermal contact resistance higher than 10^−7^ K·m^2^/W starts to degrade the thermal rectification. For a high thermal contact resistance of 10^−6^ K·m^2^/W, the maximum rectification is decreased to *RR* ∼ 32%.

As a conclusion, substantially more compact thermal storage systems can be built in the future by reducing the necessary and usually very thick insulation layer. Moreover, when such a system operates in a high-frequency regime and is adapted to, e.g., a time varying or fluctuating heat source, the difference between the final charging and cooling temperature will become smaller. It remains an open question whether such a system could be tailored even without the insulation and without substantial losses. This opens up further research frontiers that need to be addressed in the future.

### Conclusion and prospects

In summary, we present the thermal rectification properties of a thermal diode design based on a multilayer structure made of PCMs and PIMs, by means of FEM simulations. The highest *RR*s are found to be 96% and 119% for the 2-PCM/PIM and 3-PCM/PIM diodes, respectively, when *T*_*source*_ = 500 K and *T*_*sink*_ = 300 K. The temperature dependency of the *RR*s is also determined, obtaining a maximum value of 106% and 136% for 2-PCM/PIM and 3-PCM/PIM when *T*_*source*_ = 395 K and *T*_*source*_ = 472 K, respectively (*T*_*sink*_ = 300 K). These thermal *RR*s are ∼50–80% larger than in simple PCM/PIM junctions, like Nitinol (PCM) and graphite thermal diode (PIM) (Garcia-Garcia and Alvarez-Quintana, 2014). Moreover, the existence of multiple layers of PCMs and PIMs allows better control of the temperature gradients across materials, which facilitates the transition between high and low thermally conductive PCM phases at certain range of temperatures. As a result, the thermal conductivity of the 3-PCM/PIM thermal diode in the forward direction holds two plateau values at different temperatures, which qualifies this device as a multistate thermal regulator. Beyond their exceptional thermal rectification performance, the realistic material sizes and properties considered in this model make the fabrication of the thermal diode feasible. However, experimental investigation of these devices is required to validate the models developed. Finally, these thermal diodes are key enablers of new technology advances related with energy management and storage. We analyze their potential use for the storage of waste or renewable heat. The implementation of these devices allows retaining of considerably more heat during the cooling process of the energy storage element than if they were not present. They could be used to develop more compact thermal storage systems as well as for management of heat fluxes from renewable and waste heat sources, or even in processes related to the coupling of sectors (power-to-x). Overall, these thermal diodes represent new opportunities for more efficient energy processes, which are essential for our sustainable future.

## STAR★METHODS

### Key resource table

**Table:** 

RESOURCE	SOURCE	IDENTIFIER
**Software and algorithms**		

COMSOL Multiphysics 5.3	COMSOL Inc	https://www.comsol.com/
Python 3	Python Software Foundation	https://www.python.org/
Odeint	Python Software Foundation	https://www.python.org/
Origin Pro 2019b	OriginLab Corporation	https://www.originlab.com/

### Resources availability

#### Lead contact

Further information and requests for resources should be directed to and will be fulfilled by the lead contact, Dr. Miguel Muñoz Rojo (m.munozrojo@utwente.nl)

#### Materials availability

This study did not generate new unique materials.

#### Data and code availability

• All data reported in this article will be shared by the lead contact upon request
• This study does not report original code
• Any additional information required to analyze the data reported in this study is available from the lead contact upon request.

### Method details

The computational analysis presented in this study was performed by means of finite element modeling (FEM) in the software COMSOL Multiphysics. First, we determined the geometry of our structure in a rectangular shape as explained in Section S1. Second, we selected reported material properties for defining the characteristics of the individual layers (see Section S2). We analyzed different material configurations when applying a temperature gradient along the structure (see Section S3). For the FEM analysis, we used a predefined coarse mesh with a minimum element size of 2.4 nm and a maximum element size of 120 nm (see Section S4). To evaluate the different material configurations on the basis of their *RR*s, we used Equation 1. The 2D calculation in our COMSOL model is based on Fourier's law, as stated in Equation 2. Within the software, we extracted the conductive heat flux and the temperature from the results of the calculations (see Section S5). On the basis of these results and the temperature gradient, we calculated the thermal conductivity of the most relevant configurations by applying the definition of the thermal resistance (see Section S6). For the application model, we used polynomial fits created in the software Origin to obtain a mathematical expression for the used material properties. We solved the differential equation as described in Section 4 and S9 by using the odeint solver in Python 3 (see Section S9).

For an elaborated description of the above described methods, please have a look in the related sections of the supplemental information.

## Footnotes

Supplemental information can be found online at https://doi.org/10.1016/j.isci.2021.102843.
