# Supplementary material for: Thermal rectification in multilayer phase change material structures for energy storage applications
Source: iScience. 2021 Jul 10;24(8):102843. doi: 10.1016/j.isci.2021.102843 (PMC8353506; doi:10.1016/j.isci.2021.102843)
Supplement: Document S1. Figures S1–S10 and Tables S1–S4 [file mmc1.pdf]

iScience, Volume *24*

Supplemental information

Thermal rectification in multilayer phase change material structures for energy storage applications

Timm Swoboda, Katja Klinar, Shahzaib Abbasi, Gerrit Brem, Andrej Kitanovski, and Miguel Muñoz Rojo

Supporting Information: Thermal rectification in multilayer phase change material structures for energy storage applications

Timm Swoboda[1], Katja Klinar[2], Shahzaib Abbasi[1], Gerrit Brem[1], Andrej Kitanovski[2], and Miguel Muñoz Rojo[1,*]

[1]*Department of Thermal and Fluid Engineering, University of Twente, Enschede, Overijssel 7500 AE, The Netherlands*

[2]*Faculty of Mechanical Engineering, University of Ljubljana, Ljubljana, Osrednjeslovenska, Askerceva 6, 1000, Slovenia*

*corresponding author:m.munozrojo@utwente.nl

S1. Geometry

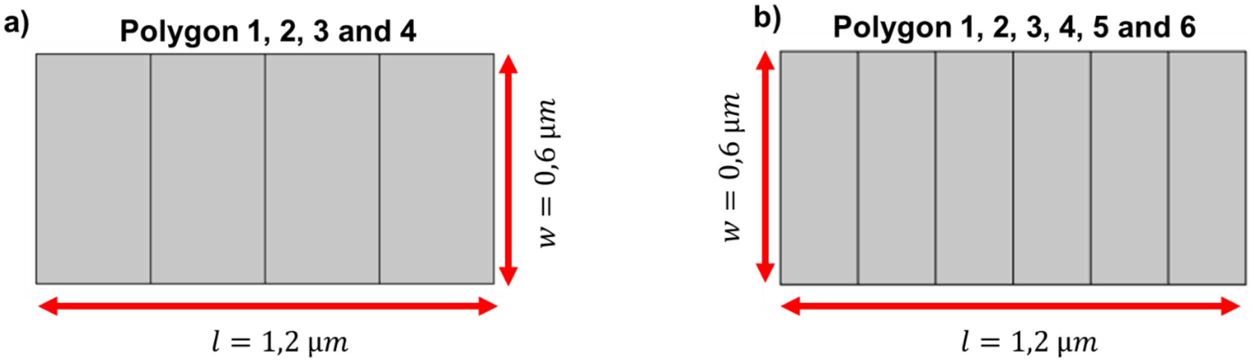

Figure S1 a) Geometry of the 2-PCM diode used in the FEM COMSOL simulations presented in this study. The geometry contains 4 equally shaped rectangular polygons, and it has a total length and width of *l* = 1.2 μm, *w* = 0.6 μm respectively. b) Geometry of the 3-PCM diode used in the FEM COMSOL simulations presented in this study. The geometry contains 6 equally shaped rectangular polygons, and it has a total length and width of *l* = 1.2 μm, *w* = 0.6 μm respectively. Related to Figure 2.

In order to compare the two examined structures properly we choose to use the same geometrical dimensions in both cases. Therefore, we combine individual rectangular shaped polygons to form structures with a total length and width of *l* = 1.2 μm, *w* = 0.6 μm respectively. The length values of each polygon are equally distributed throughout the design. Thus, in the 2-PCM diode the polygons have a length of 0.3 μm, while being 0.2 μm in the 3 PCM diode. The described geometries of the two diode design are illustrated in **Figure S1**.

S2. Material Selection

Table S1 Reported maximum and minimum thermal conductivity values (k) of the used Phase change materials (PCM), which changes at around the transition temperature T_{trans}. The PCMs are distinguished between type A and type B PCMs as they present an increase (type A) or decrease (type B) in thermal conductivity above T_{trans}.(Chen et al., (2018); Hirata et al., (2020); Oh et al., (2010)) Additionally the source of data for the two used phase invariant materials (PIM) are presented. Related to Figure 2.

Material	k [W/(m·K)] ($T<T_{trans}$)	k [W/(m·K)] ($T>T_{trans}$)	T_{trans} [K]	Type	Reference
PCMs					
Ag_2S	0.5	1	450	A	Chen et al., (2019)
Cu_2Se	0.6	0.9	400	A	Chen et al., (2019)
Ag_2Se	1.15	0.8	400	B	Chen et al., (2019)
Cu_2S	0.45	0.33	380	B	Chen et al., (2019)
Ag_2S	0.5	1.3	450	A	Hirata et al., (2020)
$Ag_2S_{0.8}Se_{0.2}$	0.45	1.2	400	A	Hirata et al., (2020)
$Ag_2S_{0.6}Se_{0.4}$	0.25	1.6	360	A	Hirata et al., (2020)
$Ag_2S_{0.4}Se_{0.6}$	0.7	2.1	350	A	Hirata et al., (2020)
$Ag_2S_{0.2}Se_{0.8}$	1.5	2	350	A	Hirata et al., (2020)
Ag_2Se	1	0.75	400	B	Hirata et al., (2020)
Ag_2Te	1	0.5	420	B	Hirata et al., (2020)
$Ag_2S_{0.05}Te_{0.95}$	1.2	0.7	400	B	Hirata et al., (2020)
$Ag_2S_{0.1}Te_{0.9}$	1.5	0.7	380	B	Hirata et al., (2020)
$Ag_2S_{0.15}Te_{0.85}$	1.8	0.8	340	B	Hirata et al., (2020)
VO_2	3.5	5.5	340	A	Oh et al., (2010)
PIMs					
Si	No phase transition				COMSOL Database
SiO_2	No phase transition				COMSOL Database

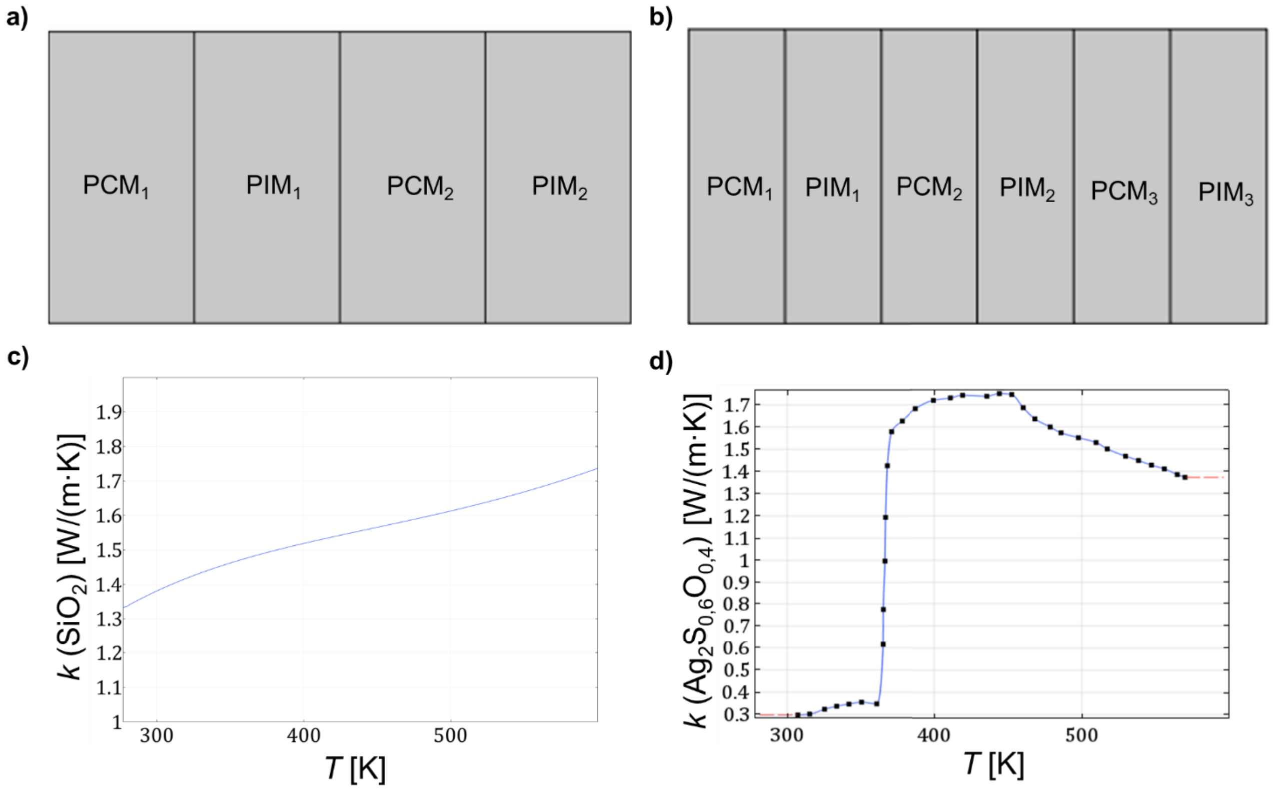

Figure S2 a) Material configuration in the 2-PCM diode structure, b) Material configuration in the 3-PCM diode structure. Thermal conductivity values used in the COMSOL simulations of c) SiO_2 (COMSOL data base) and d) $Ag_2S_{0,6}O_{0,4}$ (Hirata et al., (2020)) as a function of temperature. Related to Figure 2.

The individual polygons are connected to two types of materials, Phase invariant materials (PIMs) and Phase change materials (PCMs). The thermal conductivity values of the PIMs are taken from the COMSOL Database. We only consider PCMs with a solid to solid phase transition to completely remain in solid-state. The maximum and minimum thermal conductivity values of the included PCMs below and above the transition temperature T_{trans} are shown in **Table S1**. We extract experimental data from PCMs which present a strong change in their thermal conductivity along their phase transition from the literature. For example the graphical illustration of thermal conductivity of the COMSOL model of the PIM SiO_2 and the PCM $Ag_2S_{0,6}O_{0,4}$ are presented in **Figure S2** c) and d) as a function of the temperature.(Hirata et al., (2020)) The material properties are applied to the individual polygons of the geometry as indicated in Figure S2. In that sense we only consider PCMs for the PCM denoted polygons and only PIMs for PIM denoted Polygons. Both structures consist then of a multilayer material configuration with alternating PCM and PIM layers, as can be seen in Figure S2 a) and b).

S3. Heat Transfer model

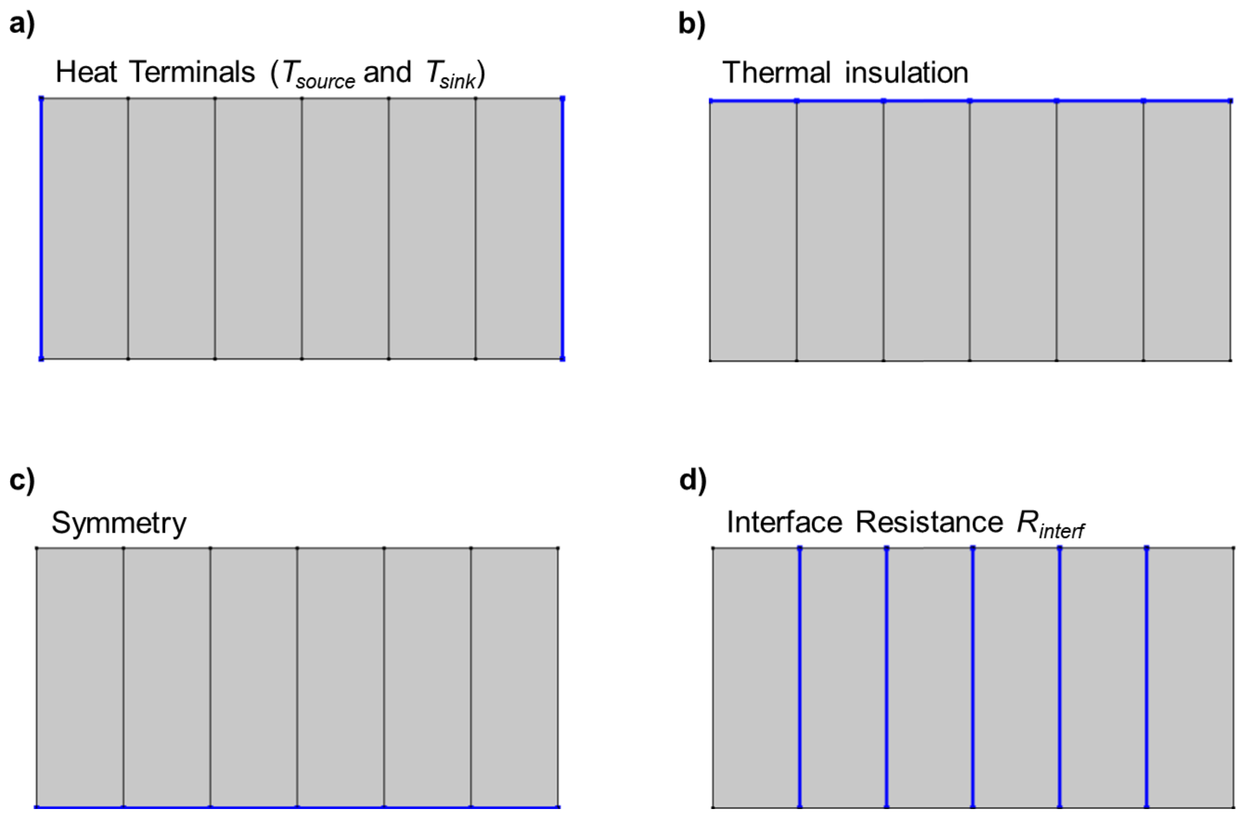

Figure S3 a) Implemented heat terminals T_{source} and T_{sink} to induce a temperature gradient ($T_{source} > T_{sink}$). In the forward heat flux calculations one of the terminals has a temperature equal T_{source} while the other one is equal T_{sink}. In the reverse case the two temperature terminals are swapped. The initial temperature is at ambient conditions, b) applied thermal insulation at the top of the structure, c) applied Symmetry at the bottom of the structure, d) applied interface resistances R_{interf} in between the different material layers. Related to Figure 3.

The thermal rectification ratio is calculated along the length of the structure. In order to create a heat flux, we apply a temperature gradient between the two heat terminals on the left and right side edge of the structure, as indicated in **Figure S3** a). One terminal is at a temperature of T_{source}, while the other one is at T_{sink} (forward direction). The position of T_{source} and T_{sink} is swapped (forward vs reverse) in order to evaluate the heat flux of the structures depending on its directionality. In our model the forward direction is always related to the heat terminal configuration which creates the higher magnitude in heat flux. The top edge of the structure is thermally insulating, to verify that the heat flux is only transported between the terminals. To create a quadratic design of the diode, symmetry is implemented at the bottom edge of the structure. The interfacial resistance is located at the transition edge between material layers (see Figure S3 d)).

S4. Mesh

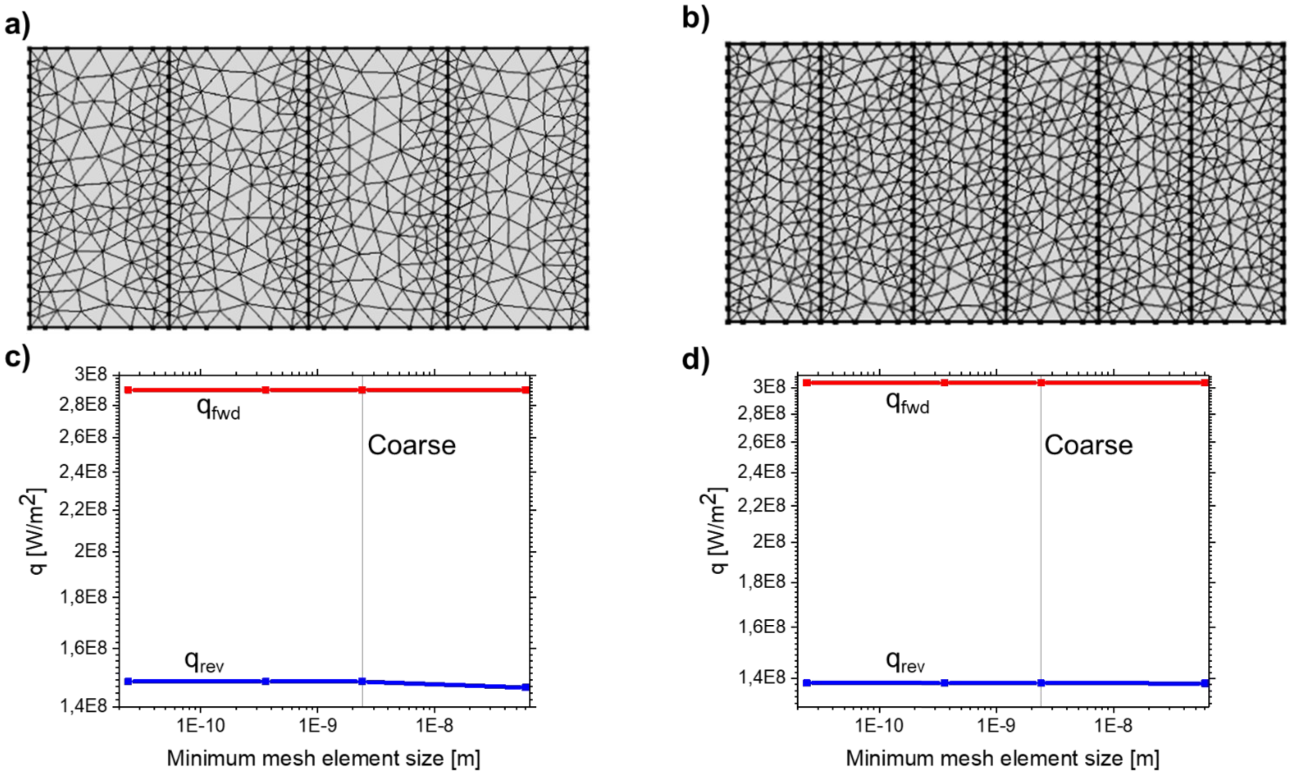

Figure S4. Mesh of the a) 2PCM structure and b) 3PCM structure used in the COMSOL simulations. (Maximum Element size 120 nm, Minimum element size 2.4 nm, Fixed number of 20 elements at every transition line). Calculated heat flux in forward (red lines) and reverse (blue lines) direction as a function of the minimum mesh element size of the COMSOL geometry of the c) 2 PCM and d) 3 PCM material configurations described in Section S2. The vertical lines in c) and d) correspond to the results obtained with the Coarse mesh presented in a) and b). Related to Figure 3.

In order to reduce the calculation time during our study, we choose to use the coarser mesh element size that possibly doesn't affect the accuracy of the results. The coarse element size is predefined ranging from minimum 2.4 nm up to a maximum of 120 nm. Additionally, we implement a fixed number of elements (20) at the edges in between materials, to increase the accuracy in transitions (**see Figure S4** a) and b)). Once we find the ideal material configuration by means of the Material sweep, we recalculate the results of the heat flux, by using a finer element size. Therefore, we adjust the fixed number of elements at the layer edges gradually. Finally, we check the results for an extremely coarse mesh without using a fixed number of elements. Figure S4 c) and d) show the calculated heat flux in forward and reverse direction of the structures presented in Section 3.1 as a function of the minimum mesh element size of the model. Here we can see that the results barely change when we use finer meshes. However, we observe a small but significant change in the results when using the extremely coarse mesh (Minimum mesh element size 60 nm). As a result, we can confirm that certain coarse meshes are sufficiently accurate and that too fine meshes are not necessary in this study since it mostly extends the computing time.

S5. Analysis

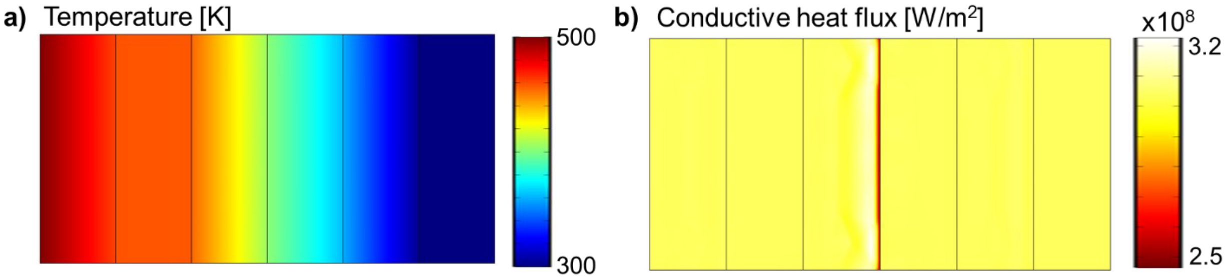

Figure S5 a) Temperature profile and b) Conductive heat flux, of the 3PCM structure described in Section 3.1 in forward direction for T_{source} = 500 K and T_{sink} = 300 K. Related to Figure 3.

We apply a material sweep to find the optimal material configuration. Therefore, we consider every possible material combination (see Table S1), related to the alternating PCM/PIM structures indicated in Figure S2 a) and b). For each case we calculate the conductive heat flux in x-direction (along the structure) using a stationary solver. Given the fact that our structures represent a series of heat resistances, the heat flux is constant along them. In order to eliminate minor fluctuations at phase transitions we determine the average heat flux of all layers. We repeat the same procedure, while reversing the temperature gradient. We then calculate the rectification ratio *RR* by using equation 1 of the manuscript. Therefore, we presuppose that $|q_{fwd}| > |q_{rev}|$ in every configuration. **Figure S5** shows the results obtained for the 3-PCM structure with the material configuration as explained in Section 3.1 in forward direction. As can be seen in Figure S5 b) the conductive heat flux slightly fluctuates in the 3rd layer. This small fluctuation is connected to a phase transition of the PCM in the 3rd layer as can be seen in Figure 3 b).

Afterwards we use the material configuration with the highest *RR* to investigate the impact of T_{source} on the rectification behavior. Therefore, we apply a parametric sweep in which we calculate *RR* for the aforementioned structure by varying T_{source} from 300 to 550 K with 1 K steps. We choose this temperature range, because the used experimental data of the PCM materials does not present values above 550 K. The results of this analysis are presented in Figure 4.

S6. Calculation of thermal conductivity

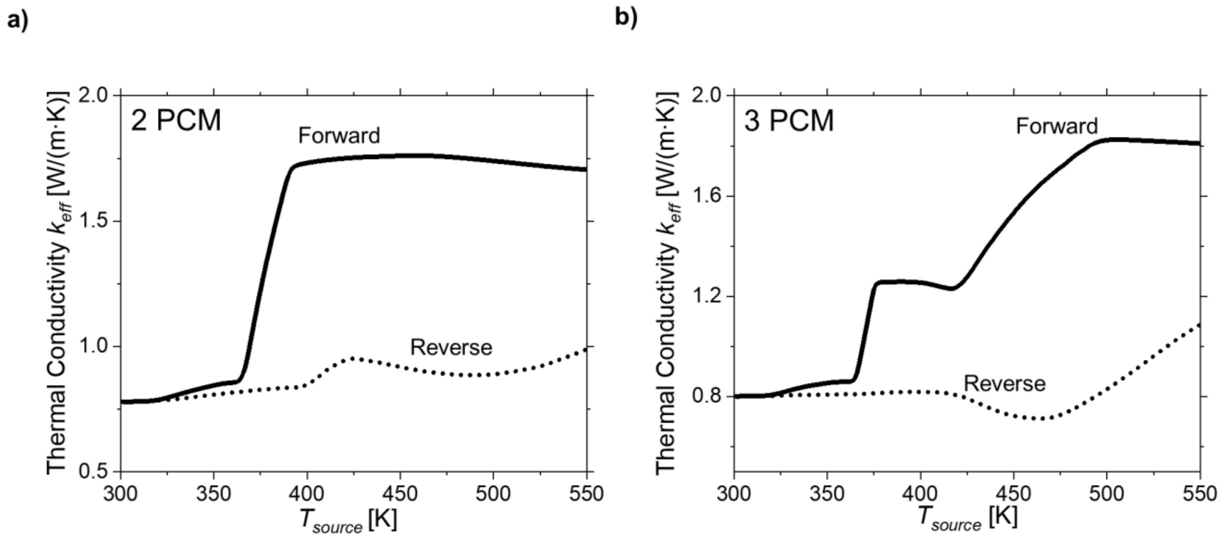

Figure S6: Effective thermal conductivity in forward (solid) and reverse (dotted) direction of the a) 2PCM diode and b) 3PCM diode configuration, presented in Section 3.1 as a function of the temperature at the heat source T_{source}. Related to Figure 3.

The effective thermal conductivity for the diode configurations is calculated by using the basic definitions of the thermal resistance $R_{thermal}$, presented in equation S1 and S2:

$$R_{thermal} = \frac{l}{k_{eff} \cdot A} \quad \text{(S1)}$$

$$R_{thermal} = \frac{\Delta T}{q \cdot A} \quad \text{(S2)}$$

In which l, A and k_{eff} correspond to the length, the cross section and the thermal conductivity of the diodes. The temperature gradient ΔT corresponds to the temperature difference between T_{source} and T_{sink}, while q is the heat flux per square area in W/m^2. By combining equation S1 and S2 we obtain an expression to calculate k_{eff}.

$$k_{eff} = \frac{l \cdot q}{\Delta T} \quad \text{(S3)}$$

We then calculate the thermal conductivity values in forward and reverse direction for the discussed diode design on base of the calculated heat flux and the in the model defined parameters. **Figure S6** displays the k_{eff} values for the diode designs of Section 3.1 as a function of the temperature at T_{source}. Therefore, the results of the parametric sweep, explained in Section S5 are used. In Figure S6 b) we can see that the thermal conductivity of the 3-PCM structure possesses three different thermal conductivity plateaus in forward direction. The thermal conductivity is low at a lower temperature, intermediately high at higher temperatures and reaches its maximum at high temperatures. This behavior presents an additionally interesting property of the diode, as it could be used as a multi-state thermal regulator. This would allow a higher degree of thermal modulation in comparison to simple thermal switches and regulators.(Swoboda et al., (2020))

S7. Validation

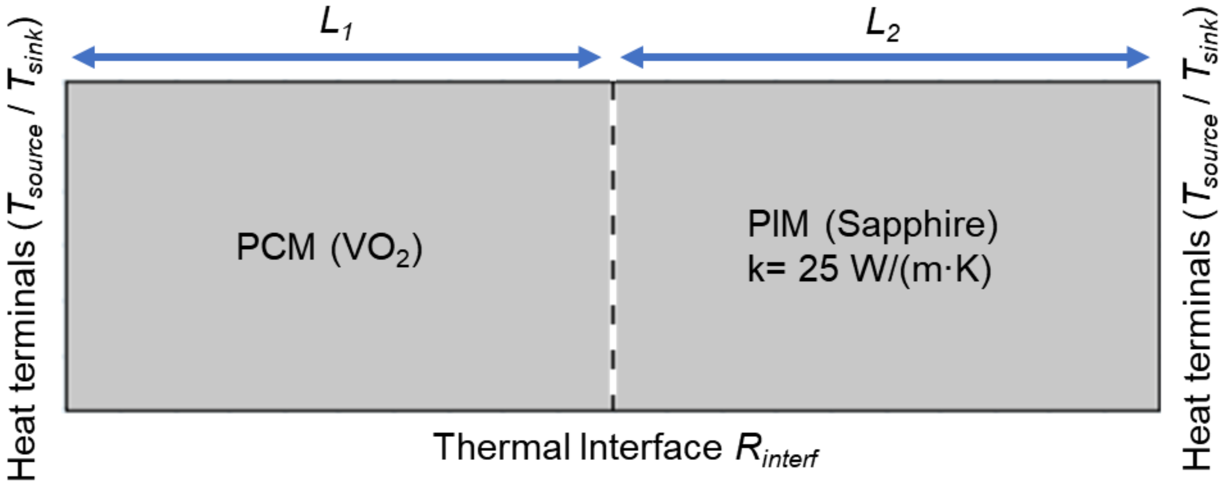

Figure S7 Geometry of the validation model. L_1 and L_2 correspond to the length of the studied material blocks. T_{source} and T_{sink} correspond to the temperature at the heat source and heat sink respectively. L_1, L_2, T_{source} and T_{sink} correspond to the same values presented in ref. (Ordonez-Miranda et al., (2018)). Related to Figure 3.

In order to validate our model we compare our simulation results with the analytical study performed by (Ordonez-Miranda et al., (2018)) on a similar diode structure. In their study, the thermal rectification ratio of a diode containing one PCM and one PIM layer is calculated. A maximum $RR_{\text{calibration}}$ = 19.7 % is obtained by using VO_2 as the PCM and Sapphire as the Non-PCM (PIM) layer. In this case $RR_{calibration}$ is calculated by using equation S4.

$$RR_{calibration} = \frac{|q_{fwd}|-|q_{rev}|}{|q_{fwd}|} \tag{S4}$$

Hereby q_{fwd} and q_{rev} are the heat fluxes in the forward and reverse direction, where $|q_{fwd}| > |q_{rev}|$. Since we use this approach as calibration for our model, we simulate (see Figure S7) the thermal rectification for the exact same diode design ($L_1 = L_2$ =1 µm, T_{source} =369.5 K, T_{sink} = 300 K, R_{interf} = 0.23 (K·mm^2)/W, k(Sapphire) = 25 W/(m·K), and k(VO_2) vs T)(Ordonez-Miranda et al., (2018)). Using equation S4, we obtain a $RR_{calibration}$ = 20.1 %, which matches very well with the results of the original study (Ordonez-Miranda et al., (2018)). One difference between the two approaches is that the authors (Ordonez-Miranda et al., (2018)) include the possible impact of thermal hysteresis (relatively small in VO_2) on *RR*. We do not include thermal hysteresis in our model, because the experimental data for the materials mentioned in Table S1 from authors (Chen et al., (2018); Hirata et al., (2020); Oh et al., (2010)) that we use do not display this effect. Anyhow, it is worth to note that this might lead to potential differences of the presented *RR* with experimentally obtained values.

S8. Influence of geometry, interfaces and number of layers in thermal rectification of PCM/PIM diodes

Table S2 Influence of different model and material parameters on key properties in PCM/PIM thermal diodes. The sign describes how the column properties change (+ increases, - decreases, 0 not affected) when the parameter in the rows is increased. Related to Figure 1.

	RR	Temperature control	Feasibility	Small-scale application	Reference
Asymmetric Geometry	+	0	-	0	(Zhu et al., (2014))
Length	+	0	+	-	
Interface Resistance	-	+/-	-	-	
Number of layers	+/-	+	0/-	0/-	
Thermal Hysteresis	-	-	-	0/-	(Ordonez-Miranda et al., (2018))

Table S2 summarizes the results of the discussion of Section 3.3. The sign in the cells describes how the column properties change when the model or material parameter mentioned in the rows rises. For instance, when the number of layers in the multilayer diode is increased the degree of temperature control increases as well. When the length of the system is increased the applicability for small-scale reduces. Some of the parameters have been previously investigated. As an example, we know that a thermal rectification has been found in an asymmetric shaped VO_2 beam.(Zhu et al., (2014)) Moreover, it has been shown that the thermal hysteresis usually leads to a decrease of the thermal rectification.(Ordonez-Miranda et al., (2018))

S9. Calculation Application model

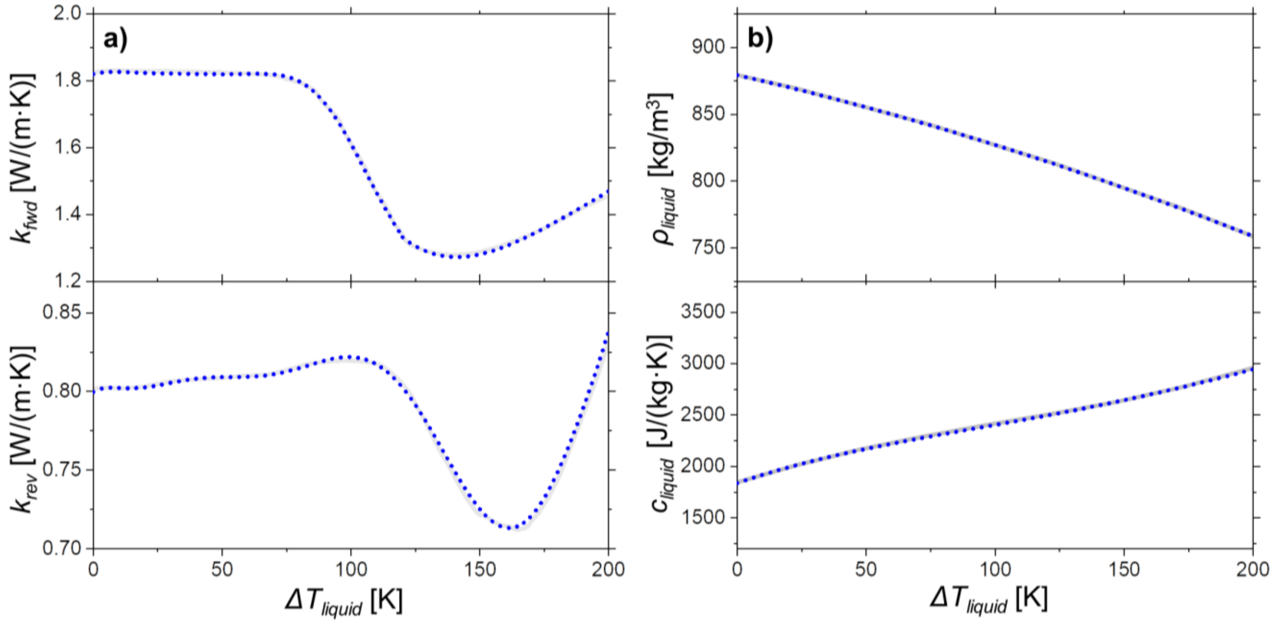

Figure S8 Simulated data of the diode behavior for the analytical application model as a function of the temperature change in the liquid reservoir ΔT_{liquid}. a) Thermal conductivity of the diode during the charging process (k_{fwd}) and the cooling process (k_{rev}). b) Heat capacity (c_{liquid}) and density (ρ_{liquid}) of the liquid Paratherm.(Paratherm, (2020)) The transparent continuous lines correspond to the reference values, the values calculated by COMSOL respectively. The blue dotted lines correspond to the fitting equations used in the analytical calculations. ΔT_{liquid} = 0 K, represents the initial temperature of the liquid T_{liquid} = 300 K. As a consequence, ΔT_{liquid} = 200 K, represents the state of the liquid at T_{liquid} = 500 K. Related to Figure 6.

Table S3 Fitted parameters of the analytical application model ($PE = V + B_1 \cdot \Delta T_{liquid} + B_2 \cdot \Delta T_{liquid}{}^2 + B_3 \cdot \Delta T_{liquid}{}^3 + B_4 \cdot \Delta T_{liquid}{}^4 + B_5 \cdot \Delta T_{liquid}{}^5 + B_6 \cdot \Delta T_{liquid}{}^6 + B_7 \cdot \Delta T_{liquid}{}^7 + B_8 \cdot \Delta T_{liquid}{}^8 + B_9 \cdot \Delta T_{liquid}{}^9$). Related to Figure 6.

PE	B1	B2	B3	B4	B5	B6	B7	B8
k_{fwd} ($\Delta T_{liquid} < 120\ K$)	0.002	-1.858 10^{-4}	4.92036 10^{-6}	9.31654 10^{-8}	-8.30838 10^{-9}	1.98051 10^{-10}	-2.26839 10^{-12}	1.26704 10^{-14}
k_{fwd} ($\Delta T_{liquid} > 120\ K$)	-0.14898	1.1272 10^{-3}	-3.67607 10^{-6}	4.4923 10^{-9}	0	0	0	0
k_{rev}	0.00113	-1.56433 10^{-4}	9.45617 10^{-6}	-2.78952 10^{-7}	4.49505 10^{-9}	-4.12891 10^{-11}	2.14614 10^{-13}	-5.86457 10^{-16}
ρ_{liquid}	-0.43363	-9.39964 10^{-4}	7.27317 10^{-7}	-1.22858 10^{-9}	0	0	0	0
c_{liquid}	8.68542	-0.05256	2.61484 10^{-4}	-3.87988 10^{-7}	0	0	0	0
	k_{fwd} ($\Delta T_{liquid} < 120\ K$)	k_{fwd} ($\Delta T_{liquid} > 120\ K$)	k_{rev}	ρ_{liquid}	c_{liquid}			
V	1.82	8.39863	0.79956	879.209	1838.94			
B9	-2.76195 10^{-17}	0	6.54495 10^{-19}	0	0			

In this section the calculation steps of the thermal diode application model are described in an elaborated way. As described in Section 4 we start with the following equations to describe the charging and cooling process of the application.

$$\text{Charging: } n_{diode} \cdot k_{fwd} \cdot A_{diode} \cdot \frac{T_{source} - T_{liquid}}{d_{diode}} = m_{liquid} \cdot c_{liquid} \cdot \frac{\delta \Delta T_{charge}}{\delta t_{ch}} \quad \text{(S5)}$$

$$\text{Cooling: } n_{diode} \cdot k_{rev} \cdot A_{diode} \cdot \frac{T_{liquid} - T_{sink}}{d_{diode}} = m_{liquid} \cdot c_{liquid} \cdot \frac{\delta \Delta T_{cooling}}{\delta t_{co}} \quad \text{(S6)}$$

The mass of the liquid is calculated by using,

$$m_{liquid} = \rho_{liquid} \cdot V_{liquid} \quad \text{(S7)}$$

In which ρ_{liquid} and V_{liquid} are the density and the volume of the liquid respectively. We choose to use the liquid Paratherm NF^{TM}(Paratherm, (2020)), as it possesses a high specific heat c_{liquid}. **Figure S8** b) shows the reference values ρ_{liquid} and c_{liquid} of Paratherm NF^{TM} as a function of the temperature rise ΔT_{liquid} of the liquid

from the initial temperature of 300 K.(Paratherm, (2020)). The mass of the liquid remains constant, thus we calculate m_{liquid} by multiplying the density by the volume of the liquid at its initial temperature of 300 K (ρ_{liquid} = 879.2 kg/m^3; V_{liquid} = 1.02·10^{-6} m^3). (Paratherm, (2020))

For our analytical calculations we fit the data by means of polynomial equations PE as described in equation S8.

$$PE = V + B_1 \cdot \Delta T_{liquid} + B_2 \cdot \Delta T_{liquid}{}^2 + B_3 \cdot \Delta T_{liquid}{}^3 + B_4 \cdot \Delta T_{liquid}{}^4 + B_5 \cdot \Delta T_{liquid}{}^5 + B_6 \cdot \Delta T_{liquid}{}^6 + B_7 \cdot \Delta T_{liquid}{}^7 + B_8 \cdot \Delta T_{liquid}{}^8 + B_9 \cdot \Delta T_{liquid}{}^9 \quad \text{(S8)}$$

with $\Delta T_{liquid}\ (t_{ch}) = \Delta T_{charge}\ (t_{ch})$ while charging and $\Delta T_{liquid}(t_{co}) = \Delta T_{charge}(t_1) - \Delta T_{cooling}(t_{co})$ while cooling. The polynomial fits of the beforementioned properties are presented in **Table S3**.

In the charging process, we assume a constant temperature of the wall at T_{source} = 500 K. The liquid is initially at room temperature ($T_{liquid,}(t_{ch}$ = 0) = 300 K) and heats up during the charging process. In the cooling process we assume that the temperature of the wall is T_{sink} = 300 K. The temperature of the liquid is then at $T_{liquid}\,(t_1)$, while heat is transported away from the liquid. In this case t_1 is the duration of the charging process, which is set to t_1 = 6 hours. The time of the cooling process is the time needed to cool the thermal storage to its original temperature in the non-diode configuration.

We implement the beforementioned modifications in the initial equations S5 and S6 to derive to equation S9 and S10.

$$\frac{k_{fwd} \cdot D \cdot (200 - \Delta T_{charge}(t_{ch}))}{\rho_{liquid} \cdot c_{liquid}} = \frac{\delta \Delta T_{charge}}{\delta t_{ch}} \quad \text{(S9)}$$

$$\frac{k_{rev} \cdot D \cdot (\Delta T_{charge}(t_1) - \Delta T_{cooling}(t_{co}))}{\rho_{liquid} \cdot c_{liquid}} = \frac{\delta \Delta T_{cooling}}{\delta t_{co}} \quad \text{(S10)}$$

We calculate the thermal conductivity of the diode in forward and reverse direction as a function of ΔT_{liquid}, by using a parametric sweep in our COMSOL model. For the charging process, we sweep T_{sink} from 300 K to 500 K for the diode in the forward direction, while keeping T_{source} = 500 K. For the cooling process we use the calculated values of the diode in reverse direction of Figure S6 b). In Figure S8 a) we plot the calculated thermal conductivity in the charge k_{fwd} and cooling k_{rev} process as a function of ΔT_{liquid}. For the non-diode configuration, we use the same thermal conductivity values in charge and in the cooling process $k_{rev} = k_{fwd}$ (k_{fwd} in both cases).

We calculate the differential equations of S9 and S10 by using the polynomial fits of Table S3 by means of a differential equation solver in Python. For the geometrical parameters we hypothesize the volume of the liquid to be V_{liquid}=1.02·10^{-6} m^3. Therefore, we assume a cubic liquid reservoir with a base length and width of 8 mm and a height of 16 mm. We choose n_{diode} = 20 and the length, width and height dimensions of the diode are set to be 1.2 µm as declared in the model in Section S1 and S3. Finally, we calculate T_{liquid} as a function of t by means of equation S9 and S10 (the results are presented in Figure 6).

The heat losses are calculated as explained in the section 4. Therefore we use the specific enthalpy values h reported in literature.("Estimating Database – EES Data," n.d.). Table S4 shows the specific enthalpy values of Paratherm used for the calculations in section 4

Table S4: Specific enthalpy h of Paratherm for the temperatures used in the calculations of section 4. Related to Figure 6.

Temperature T [K]	Specific enthalpy h [J/kg]
300	65298
312.5	88891
379.7	230291

In order to verify the calculated heat losses described in section 4 we solve the integral of $\int_0^{175000} \Delta T_{cooling}(t_{co}))\delta t_{co}$ numerically, on base of the prior obtained results of $\Delta T_{cooling}$ (175000 s ≈ 49 hours). We calculate the total heat losses ΔQ of the cooling process by means of the conduction between storage tank and wall in both cases with and without the diode, as indicated in equation S11.

$$\Delta Q = \int_0^{175000} (n_{diode} \cdot k_{rev} \cdot A_{diode} \cdot \frac{T_{liquid} - T_{sink}}{d_{diode}}) \delta t_{co} \quad \text{(S11)}$$

We assume the thermal conductivity to be constant in this temperature area (ΔT_{liquid} < 79.7 K), which is a legit approximation by considering the presented data in Figure S8 a). From Figure S8 a) we determine the thermal conductivity of the diode configuration as $k_{rev,diode}$ = 0.81 W/(m·K), and as $k_{rev,no\ diode}$ = 1.82 W/(m·K) for non-diode configuration. We then calculate the heat losses as follows:

$$\Delta Q = \frac{n_{diode} \cdot k_{rev} \cdot A_{diode}}{d_{diode}} \cdot \int_0^{175000} (\Delta T_{charge}(t_1) - \Delta T_{cooling}(t_{co}))\delta t_{co} \tag{S12}$$

$$\Delta Q_{diode} = \frac{n_{diode} \cdot k_{rev,diode} \cdot A_{diode}}{d_{diode}} \cdot \left([\Delta T_{charge}(t_1) \cdot t_{co}]_0^{175000} - \int_0^{175000} \Delta T_{cooling,diode}(t))\delta t_{co} \right) = 127.5 J \tag{S13}$$

$$\Delta Q_{no\ diode} = \frac{n_{diode} \cdot k_{rev,no\ diode} \cdot A_{diode}}{d_{diode}} \cdot ([\Delta T_{charge}(t_1) \cdot t_{co}]_0^{175000} - \int_0^{175000} \Delta T_{cooling,no\ diode}(t))\delta t_{co}) = 145.2 J \tag{S14}$$

Additionally, we can calculate the magnitude of energy difference in the liquid ΔQ using,

$$\Delta Q(\Delta T_{charge}) = m_{liquid} \cdot c_{liquid} \cdot \Delta T_{charge} \tag{S15}$$

while we average the values of the heat capacity of the liquid in the considered temperature span. After the charging process the liquid heats to 379.7 K. By using equation S15 the energy difference in the liquid is 148.9 J due to the rise in temperature (ΔT_{charge} = 79.67 K; c_{liquid} = 2076 J/(kg·K); m_{liquid} = 0.9 g). As mentioned before, the storage is cooled to its initial temperature after the cooling process in the non-diode configuration. Hence, the initial energy difference is equal to the heat losses in the cooling process $\Delta Q_{no\ diode}$ = 148.9 J. In the diode configuration the temperature in the liquid remains at 312.5 K after the cooling process. In that consequence the energy difference in regard to the initial state is 21.4 J (ΔT_{charge} = 12.5 K; c_{liquid} = 1890 J/(kg·K); m_{liquid} = 0.9 g). By subtracting the remaining energy difference from the energy difference after charging we determine the heat losses of the liquid in the cooling process for the diode configuration ΔQ_{diode} = 127.5 J. As expected, these results match very well with the results obtained in Section 4 of the manuscript. Hereby we calculate the heat losses by means of the enthalpy difference in the liquid.

S10. Cycling of thermal storage system

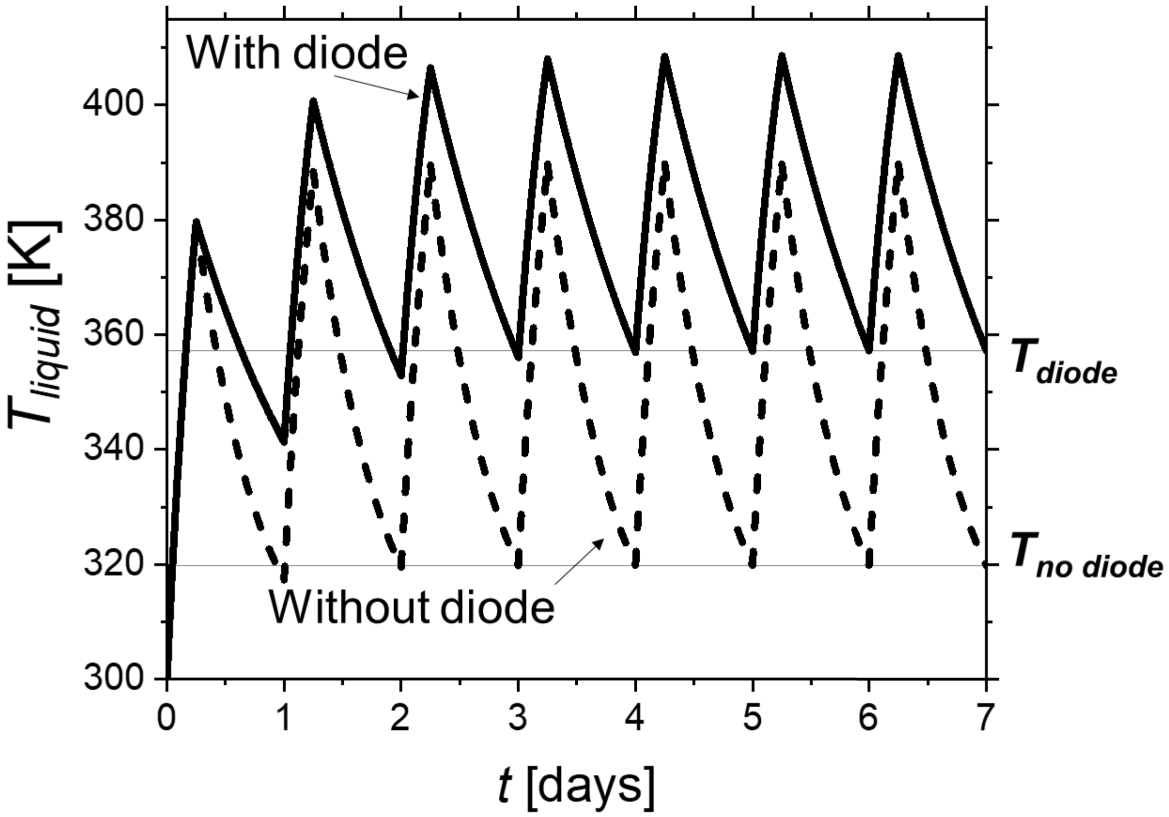

Figure S9: Temperature of the liquid heat reservoir of the energy storage element during charging and cooling as a function of the cycling time. After a few days the charging and cooling process reach an equilibrium state. In the equilibrium state the temperature after cooling is $T_{no\ diode} \approx 320$ K and $T_{diode} \approx 357$ K without and with diode respectively. Related to Figure 6.

With the intention of evaluating the long term performance of the thermal storage element we investigate the charging and cooling process for several cycles. Therefore, one cycle includes the charging and cooling process which cover one single day (t_{ch} = 6 hours and t_{co} = 18 hours). The cycle is repeated 7 times, in which every cycle starts after the end of the previous one. After one week both cycling processes (with and without diode) reach an equilibrium state. After cooling the temperature stabilizes at a temperature of T_{diode} = 357 K and $T_{no\ diode}$ = 320 K for the configuration with and without diode respectively. As a result of that we are able to store more energy in the heat storage element, using the diode configuration when the steady state is reached.

S11. Integration of thermal contacts

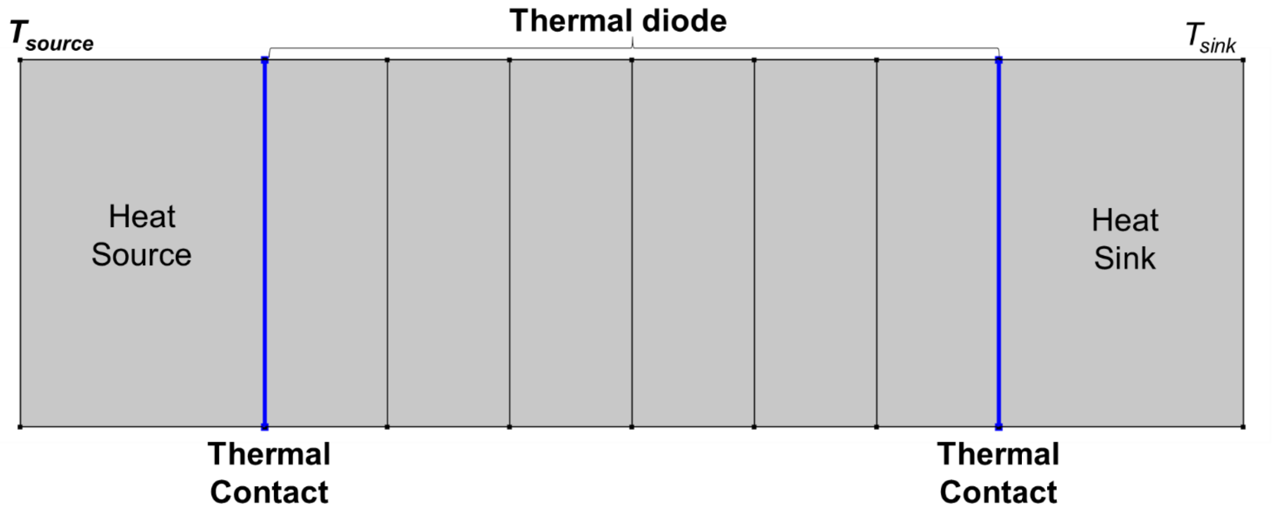

Figure S10: Thermal diode configuration with integrated thermal contact between the heat source and heat sink and the thermal diode. In this case the temperature gradient between the source T_{source} and T_{sink} is applied between the outer edges of the structure. Related to Figure 5.

In the original model stated in section 3 we assume a constant temperature at the edges of the diode. However, when implementing the diode in between a heat source and heat sink of a certain material the rectification performance would be affected due to the existence of thermal contact resistances between the thermal diode and the heat terminals. In order to evaluate the impact of the thermal contact resistance on the diode performance we recalculate the thermal properties of the 3 PCM diode presented in Section 3.1. The thermal contact resistance usually varies from 10^{-7} K·m^2/W to 10^{-5} K·m^2/W.(Fenech and Rohsenow, (1963)) We reevaluate the thermal rectification for two cases in which the thermal contact resistance is equal to 10^{-7} K·m^2/W and 10^{-6} K·m^2/W respectively. We adjust T_{source} and T_{sink}, so that the temperature gradient in thermal diode itself is as close as possible to the values stated in section 3. For a thermal contact resistance of 10^{-7} K·m^2/W we observe a slight shift in the rectification ratio and temperature gradient to RR = 101 % at T_{source} = 490 K and T_{sink} = 290 K. However, the thermal rectification and temperature gradient shift strongly for a thermal contact resistance of 10^{-6} K·m^2/W that lead to RR = 32 % between T_{source} = 570 K and T_{sink} = 200 K. We can conclude that an additional increase of the thermal contact resistance would lead to an even bigger shift of the thermal rectification properties. As a result, we conclude that a high thermal contact resistance will result in a significant change of the thermal rectification properties.
